# High prevalence of “non-dipping” blood pressure and vascular stiffness in HIV-infected South Africans on antiretrovirals

**DOI:** 10.1371/journal.pone.0185003

**Published:** 2017-09-20

**Authors:** M. S. Borkum, J. M. Heckmann, K. Manning, J. A. Dave, N. S. Levitt, B. L. Rayner, N. Wearne

**Affiliations:** 1 Department of Nephrology and Hypertension, University of Cape Town/Groote Schuur Hospital, Cape Town, South Africa; 2 Division of Neurology, Department of Medicine, University of Cape Town/Groote Schuur Hospital, Cape Town, South Africa; 3 Department of Medicine, University of Cape Town, Cape Town, South Africa; 4 Department of Endocrinology and Metabolism, University of Cape Town/Groote Schuur Hospital, Cape Town, South Africa; Imperial College London, UNITED KINGDOM

## Abstract

**Background:**

HIV-infected individuals are at increased risk of tissue inflammation and accelerated vascular aging (‘inflamm-aging’). Abnormal diurnal blood pressure (BP) rhythms such as non-dipping may contribute to an increased risk of cardiovascular and cerebrovascular events in HIV infected individuals. However, little data exists on ambulatory blood pressure (ABP) and measures of vascular stiffness in the black African HIV infected population.

**Methods:**

This is a cross-sectional analysis of otherwise well, HIV infected outpatients on ART for >5 years. Study assessments included: 24hr ABP monitoring, pulse wave velocity (PWV) and central aortic systolic pressure (CASP) using a AtCor Medical Sphygmocor device, fasting lipogram, oral glucose tolerance test, high-sensitivity C-reactive protein (hsCRP) and anthropometric data. Patients completed a questionnaire of autonomic symptoms. CD4+ counts and viral loads were obtained from the National Laboratory results system.

**Results:**

Sixty seven black participants were included in the analysis of whom 91% (n = 61) were female with a mean age of 42.2 ± 8.6 years. The median duration on ART was 7.5 years (IQR = 6–10), 84% were virally supressed and the median CD4 count was 529.5cells/mm^3^ (IQR = 372.0–686.5). The majority (67%) were classified as overweight and 76% had an increased waist circumference, yet only 88% of participants were normotensive. A hsCRP level in the high cardiovascular risk category was found in 68% of participants. The prevalence of non-dipping BP was 65%. Interestingly, there was no association on multivariable analysis between dipping status and traditional risk factors for non-dipping BP, such as: obesity, autonomic dysfunction and older age.

**Conclusion:**

This relatively young cross-sectional sample of predominantly normotensive, but overweight black women on effective ART >5 years showed: a high prevalence of non-dipping BP, inflammation and vascular stiffness. Causality cannot be inferred but cardiovascular risk reduction should be emphasized in these patients.

## Introduction

The wide spread roll-out of antiretroviral therapy (ART) has resulted in HIV-infected individuals living longer.[1] However, increased survival on ART has been associated with a rise in chronic co-morbidities such as cardiovascular, renal and metabolic diseases.[2] HIV infection is increasingly recognized as an independent cardiovascular risk factor, although these data are mainly from developed countries.[3–5] Age-related co-morbidities seem to occur at a younger age in HIV-infected patients and this accelerated aging may relate to: chronic ART usage, immune activation and inflammation (‘inflamm- aging’).[1,3]

HIV infection is characterized by chronic immune dysregulation despite effective ART.[6] High sensitivity C-reactive protein (hsCRP) remains elevated in patients on ART, possibly signifying persistent immune and endothelial activation.[7–9] Levels of hsCRP, interleukin-6 (IL-6) and D-dimer have been shown to predict mortality in black South African HIV-infected patients on ART with preserved CD4+ counts.[10] Furthermore, hsCRP and IL-6 are considered to be potential biomarkers for assessing cardiovascular risk in both HIV-infected and uninfected patients.[8,11,12]

Other measures of cardiovascular risk include: pulse wave velocity (PWV), central aortic systolic pressure (CASP) and the absence of nocturnal dipping of blood pressure (BP) on 24 hour ambulatory BP monitoring (ABPM). An overnight drop in systolic BP of less than 10% of the daytime systolic BP is a marker of increased cardiovascular risk in hypertensive and normotensive individuals.[13] Studies have found that abnormal ABPM may be more common in HIV-infected individuals. However, these studies are largely confined to European cohorts.[14] Information is also scant on markers of vascular stiffness, PWV and CASP in sub-Saharan Africa. Mixed data exists on whether treated HIV infection is associated with increased PWV and CASP.[15–17]

Most studies suggest a greater prevalence of nocturnal non-dipping BP in HIV-uninfected African Americans compared to their white counterparts.[18,19] However, ABPM patterns have been scarcely described in HIV-infected or uninfected black African populations. A South African study, comparing ABPM in black and Indian-ancestry medical students, revealed less nocturnal dipping and a higher left ventricular mass in the black students.[20] In the only other known ABPM study in black Africans, of 78 HIV status unknown Nigerian hypertensive subjects, mean age 53 ± 13 years and body mass index (BMI) 30.0kg/m^2^, non-dipping BP was seen in a high percentage of participants, 47.6% of males and 38.9% of females.[21]

A South African study previously reported a trend towards a higher frequency of non-dipping BP in HIV-infected (82%) compared to HIV-uninfected participants (53%) (*p* = 0.05, odds ratio = 3.56). In this pilot study the short- term use of ART did not restore nocturnal BP dipping.[22] This high prevalence of non-dipping BP may be explained by ‘inflamm- aging’, autonomic and baroreceptor dysfunction, poor sleep quality or social stressors.[14] Here we examine the long-term effect of ART on ABPM and vascular stiffness determined using CASP and PWV in a longitudinal cohort of HIV-infected patients on ART.

## Methods

### Study population

Ethics approval was granted for the study by the University of Cape Town, Human Research Ethics Committee (HREC REF 397/2014). This study included: black South Africans, over 18 years old, who had received more than 5 years of ART and had participated in one of two previous ‘MCHAART’ (Metabolic Complications of Highly Active AntiRetroviral Therapy) studies. All participants were recruited from the Crossroads Community Health Centre, Cape Town. Approximately 1/3 formed part of a prospective, longitudinal study investigating the metabolic and neuropathic complications after starting ART in HIV-infected South Africans.[23,24] The remaining 2/3 were from a similar, although cross-sectional, cohort of participants on first-line ART.[25] Participants were recruited telephonically, from August 2015- November 2016, and provided written informed consent. Exclusion criteria from entering the original MCHAART studies were as follows: known diabetes (prior to ART), acute opportunistic infection, abuse of alcohol or illicit substances, pregnancy or corticosteroid use and known liver or renal disease. For this study the same exclusion criteria were adhered to.

#### Data collection

Study assessments were conducted over 2 consecutive days. Patient visits were commenced between 08h00-09h00 on both days. ABPM was set up by a trained nurse with an oscillometric device (SpaceLabs Medical Inc, WA, USA). The devices are callibrated and serviced annually and appropriately sized cuffs were used for each participant. BP and heart rate were recorded every 20 minutes during the day (06:00 to 22:00) and every 30 minutes at night (22:00 to 06:00). Participants were asked about hours slept and night shift work to ensure an accurate interpretation of results. Highly discrepant results were discarded, only <10% of readings were unusable in each case. The AtCor Medical Sphygmocor device uses a standard brachial cuff, femoral artery cuff and a tonometer to non-invasively measure CASP and PWV.[26] After the patient had rested for 15 minutes, three readings were taken for each variable and the calculated average was used for analysis.

Each patient had the following blood tests: hsCRP, oral glucose tolerance test (OGTT), fasting lipogram, serum creatinine and uric acid. Autonomic dysfunction was determined on history using the Survey of Autonomic Symptoms (SAS)[27] and clinically using R-R interval change and orthostatic hypotension as described in the Ewing classic battery.[28] A full medical history including habits, co-morbid disease, pregancy, medication usage and ART history was taken. Patient height, weight and waist circumference were measured by one investigator. All patients were clinically examined by the same specialist physician (MB) to exclude any intercurrent illness.

The SAS is a well- validated tool to assess the presence and severity of autonomic symptoms.[27] This survey consists of 11 items in women and 12 in men. Each item is rated with an impact score ranging from 1 (least severe) to 5 (most severe) to determine the total impact score (TIS). In individuals younger than 65 years, a SAS total of more than 3 symptoms provide a greater than 90% specificity and 65% sensitivity in determining autonomic dysfunction, and a SAS TIS of more than 7 provides greater than 90% specificity and greater than 60% sensitivity in determining severe symptoms of autonomic dysfunction.[28]

**Fig 1:**
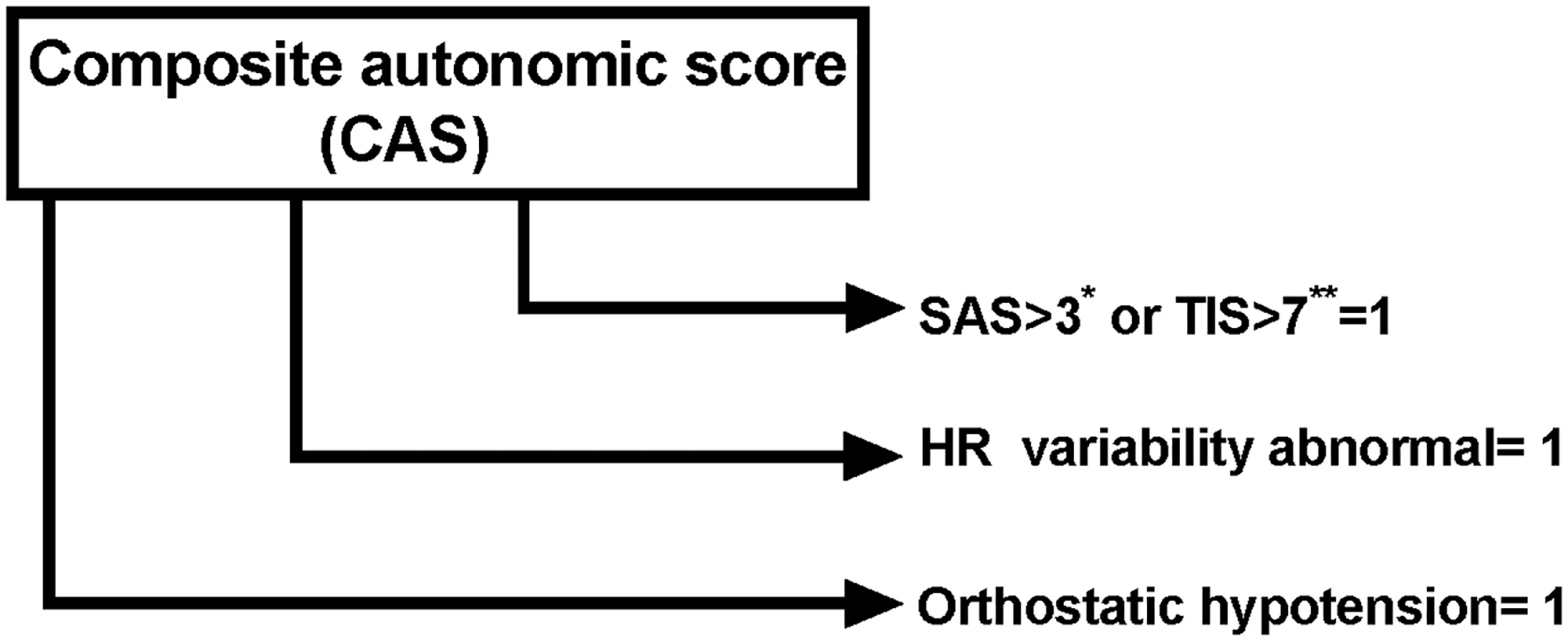
Composite autonomic score. *Survey of autonomic symptoms[27]. **Total impact score[27].

A composite autonomic score (CAS) was used to assess autonomic dysfunction (Fig 1). The maximum score was 3 and comprised of: 1. R-R interval abnormality (0 = normal or 1 = abnormal) 2. Abnormal orthostatic hypotension (0 = normal or 1 = abnormal) and 3. Critical number of autonomic symptoms (i.e. 3 or less = 0; 4 or more = 1 or total impact score (TIS) >7 = 1).

#### Statistical analysis

The data were entered into a Microsoft excel database before being exported to Stata (version 14.1, Stata Corp, College Station, Texas) for analysis, while graphical illustration of data was conducted in Prism software. Descriptive statistics and analyses were performed according to whether the variable was continuous or categorical. Depending on distribution of continuous data, variables were expressed as mean values ± standard deviation (SD) or median values with interquartile range (IQR), and compared using the Students t-test (normally distributed data) or Wilcoxon Rank Sum (non-normally distributed data). Categorical data were expressed as frequencies and percentages and compared using the χ^2^ test or Fisher exact test.

A log-binomial model was used to determine prevalent risk factors and associations for non-dipping BP. Univariate log-binomial modeling was used to explore associations and estimate unadjusted prevalence ratios for clinically relevant covariates. Covariates with suggestive association (p<0.25) with the outcome and/or had significant clinical relevance were retained in multivariable model. Unadjusted and adjusted prevalence ratios were presented with 95% confidence intervals (CI), and a p<0.05 was considered statistically significant where appropriate.

#### Definitions

Viral suppression was assessed using the HIV research network definition of a viral load of <400 cps/ml. This definition is used to eliminate most cases of apparent viremia caused by blips or assay variability.[29]

The American Heart Association and Centre for Disease Control classification of cardiovascular risk according to hsCRP level was used. A hsCRP of >3mg/L represents high risk, 1-3mg/L intermediate risk and <1 mg/L low risk.[30]

Estimated glomerular filtration rate (eGFR) was estimated using the CKD-EPI equation.[31] Normal renal function was defined as a GFR >60mL/min per 1.73^2^. Microalbuminuria was defined as albumin excretion of 30–300μg/mg of creatinine in 2 spot samples taken on separate days.

Hypertension was defined as a SBP ≥140 mmHg or diastolic BP (DBP) ≥90 mmHg, in accordance with the 2014 South African hypertension guidelines.[32] Non-dipping BP was defined as a nocturnal reduction of SBP <10% and a reverse dipper was defined as a nocturnal BP rise.[13] No large scale normative values for PWV and CASP are available for black South Africans.

The presence of 3 of 5 of the following risk factors constituted a diagnosis of the metabolic syndrome: triglycerides ≥1.7mmol/l, HDL <1.0mmol/L in males and <1.3mmol/L in females, elevated BP (as previously defined), waist circumference defined by African thresholds (≥94cm in males and ≥80cm in females) and an elevated fasting glucose (>6.1mmol/L).[33] WHO criteria were used for classifying impaired glucose tolerance and diabetes based on an OGTT.[34]

## Results

Of the 92 potential participants, 10 were not contactable. Of the remaining 82 participants, 69 agreed to be tested although two were subsequently excluded from the analysis as one participant had defaulted ART and was abusing alcohol and the other had only been on ART for 2 years. Of the 67 remaining participants one patient defaulted the second day of testing and therefore only the medical history and anthropometry were obtained for this patient and not the 24hr ABPM and blood tests results.

Mean age was 42.2 years and 91% (n = 61) were female (Table 1). Median duration on ART was 7.5 years and median CD4 count was 529.5cells/mm^3^ and 84% of participants were virally suppressed. More than 67% of the patients (n = 44) were classified as overweight (BMI >25kg/m^2^) or obese (BMI >30kg/m^2^).^(33)^ 80% (n = 48) of the women and 2 of 6 male participants had an increased waist circumference. Mean day systolic BP was 120mmHg (SD ± 17) and mean day diastolic BP was 78mmHg (SD ±13). The 10 smokers used less than 5 cigarettes per day.

**Table 1:** Demographic, anthropometric and HIV-infection information.

Descriptive characteristics (n = 67)	
**Age (years) mean ± SD**	42.2 ± 8.6
**BMI (kg/m^2^) mean ± SD**	
**Men (n = 6)**	22.6 ± 8.1
**Women (n = 61)**	27.6 ± 2.6
**Smoking n(%)**	10 (15)
**Waist circumference (cm) mean ± SD**	
**Men (n = 6)**	87.7 ± 15.3
**Women (n = 61)**	92.3 ± 14.7
**CD4 (cells/mm^3^) median (IQR)**	529.5 (372.0–686.5)
**Duration on ART (years) median (IQR)**	7.5 (6–10)
**Viral supression n(%)**	56 (84)

**Fig 2:**
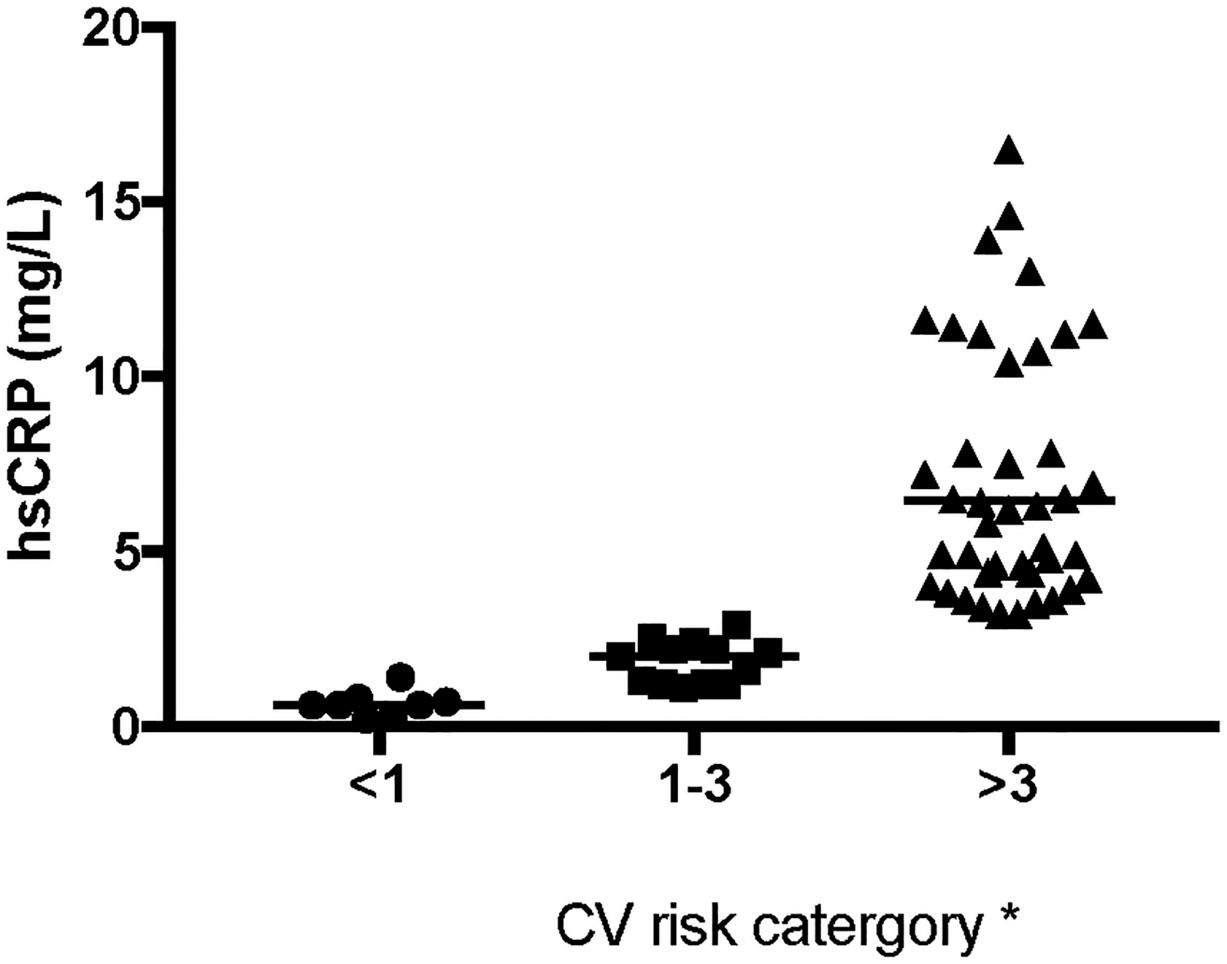
hsCRP levels in study sample. *Risk categories: <1 low risk, 1–3 medium risk, >3 high risk, Median value is represented by the horizontal line in each column. 5 data points not shown as outside axis limits (20mg/L). 2 of these samples had levels >33.1mg/L, however due to variable assay ceiling reliability were captured as 34mg/L.

A hsCRP >3mg/L was measured in 45 (68%) of participants and 13 (19%) had a hsCRP between 1-3mg/L. Only 8 (12%) participants had hsCRP levels in the low risk category (<1mg/L) (Fig 2). An elevated waist circumference was significantly correlated with increased hsCRP level (p = 0.011).

CAS results were quantified as follows: twenty six patients (39%) had a CAS of 0, twenty nine (43%) had a CAS of 1, six (9%) had a CAS of 2, five (7%) had a CAS of 3 and one patient didn’t complete testing. Of the eleven cases with a CAS ≥2, 8 had a postural drop and 9 had RR interval abnormalities.

**Fig 3:**
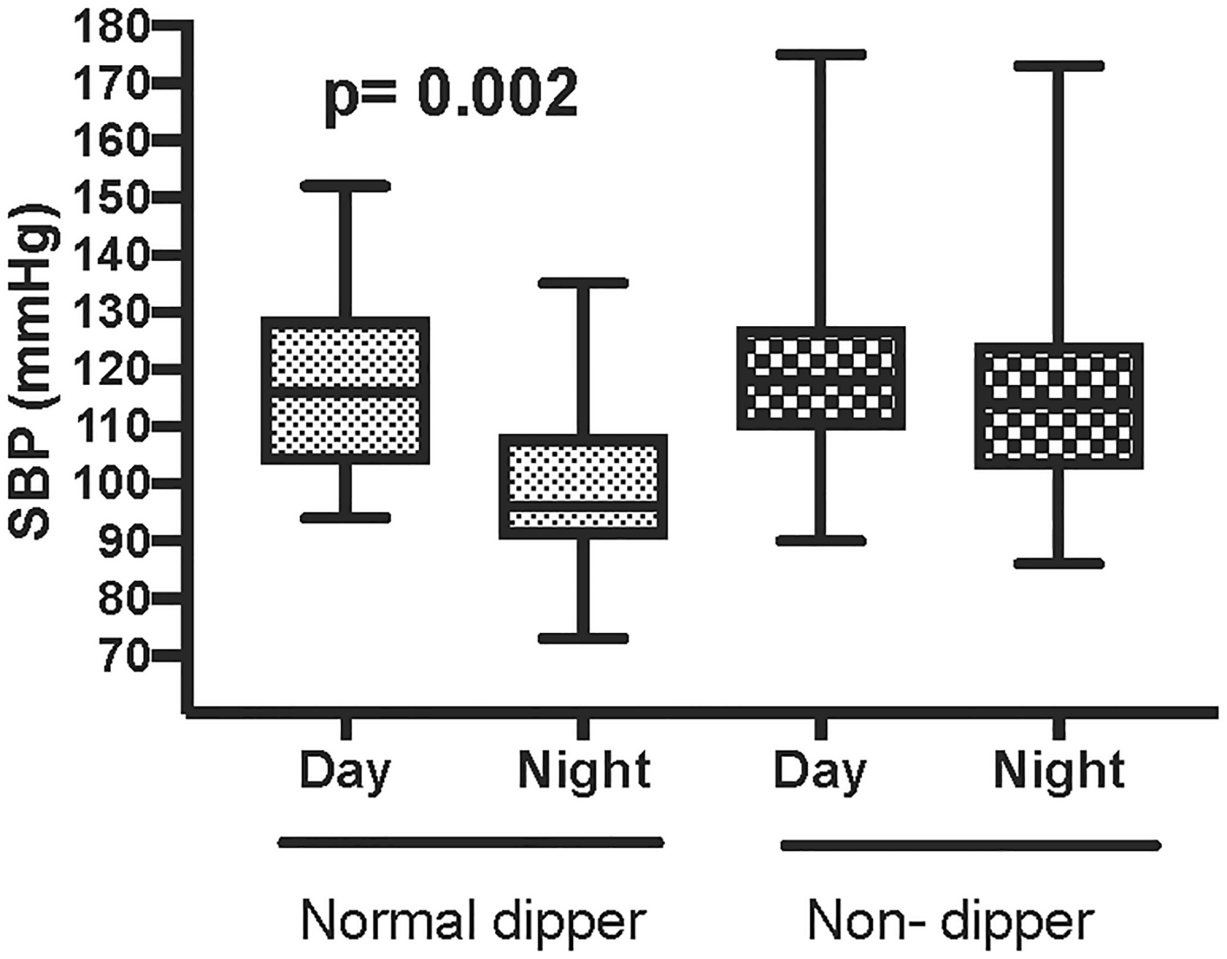
Day and night systolic BP among dippers (n = 23) and non-dippers (n = 43).

Thirteen patients (20%) had 3 or more features of the metabolic syndrome. There were 8 known hypertensives, 6 were on 1 antihypertensive agent and 2 were on 2 agents. Only 4/67 had dysglycaemia; one participant was known to have previously developed diabetes after ART initiation, 1 was newly diagnosed with diabetes and 2 with impaired glucose tolerance. Only 2 patients from the whole cohort had an abnormal eGFR and 5 had microalbuminuria. On 24 hour ABPM 65% (n = 43) of the patients were systolic BP non-dippers (Fig 3) and 1 of these patients was a reverse BP dipper. No significant differences in cardiovascular risk factors were found between BP dippers and non-dippers (Table 2).

**Table 2:** Comparison of cardiovascular risk factors between BP dippers (n = 23) and non-dippers (n = 43).

Variable	Dipper	Non- dipper	p value
Age years, mean (±SD)	43.04 (8.85)	41.83 (8.56)	0.49^○^
**BMI category, n(%)**			0.59^◾^
**Normal weight**	10 (23)	13 (57)	
**Overweight**	7 (16)	15 (35)	
**Obese**	6 (14)	15 (35)	
**Waist cm, mean (±SD)**	90.50 (15.35)	92.58 (14.50)	0.56^○^
SBP* mmHg, mean (±SD)	119.87 (23.27)	127.77 (18.12)	0.14^○^
DBP* mmHg. mean (±SD)	75.52 (15.69)	79.37 (13.56)	0.21^○^
**CAS (autonomic dysfunction) Mean (±SD)**	1.04 (0.93)	0.74 (0.85)	0.16^○^
**Microalbuminuria**	1	4	0.73^○^
CD4 cells/mm^3^, mdn (IQR)	569 (517–822)	480 (365–647)	0.13^○^
**Viral suppression, n(%)**	19 (86.36)	35 (83.33)	1.00^○^
hsCRP range, n(%)			0.79^◾^
<1	2 (9)	6 (14)	
1–3	4 (17)	9 (21)	
>3	17 (74)	28 (65)	

*Mean daytime pressures from ABP monitoring

^○^Two sample Wilcoxon rank-sum test

^◾^Fisher’s exact test

No CASP standard values are available in black Africans. By comparison to normative values for PWV in a small, healthy urban South African cohort, with a similar mean BMI (23.9kg/m^2^ in males and 27.8kg/m^2^ in females), the majority of our sample (30–49 age group) had higher mean PWV values compared to age-matched values (Table 3).[35]

**Table 3:** Mean PWV (ms^-1^) in the South African (SA) black subpopulation: HIV-infected participants on ART compared to HIV-uninfected controls[35].

Age (years)	Shiburi *et al*, SA black controls [35] Mean (SD), (n)	Study sample, SA black HIV-ART Mean (SD), (n)
**<30**	5.0 (1.2), (84)	5.6 (0.5), (7)
**30–49**	6.2 (1.2), (52)	6.8 (1.5), (47)
**≥50**	7.6 (2.5), (23)	7.5 (2.2), (11)

On univariate analysis only CD4 <500 cells/mm^3^ was a statistically significant risk factor for non-dipping BP [p = 0.04] (Table 4). Traditional risk factors for non-dipping BP such as obesity, autonomic dysfunction (as measured by CAS) and age were not significant. The association between a CD4 <500cells/mm^3^ and non-dipping BP was not maintained in multivariate analysis after adjustment for age and waist circumference. There were no other significant associations observed in the multivariate analysis. CAS, despite p = 0.12, could not be included in the multivariate analysis as it prevented convergence of the model.

**Table 4:** Unadjusted and adjusted PR for prevalent risk factors associated with non-dipping BP.

Variable	Univariate*		Multivariate**	
	Unadjusted PR (95% CI)	p value	Adjusted PR (95% CI)	p value
Age >45 years	0.74 (0.50–1.11)	0.15	0.85 (0.59–1.22)	0.38
BMI				
>25kg/m^2^	1.21 (0.76–1.91)	0.42		
>30kg/m^2^	1.26 (0.81–1.98)	0.31		
Waist circumference	1.40 (0.83–2.36)	0.20	1.30 (0.81–2.10)	0.28
Autonomic dysfunction (CAS)	0.48 (0.19–1.21)	0.12	**-**	**-**
CD4 <500 cells/mm^3^	1.43 (1.02–1.99)	0.04	1.31(0.94–1.81)	0.11
Viral suppression	0.93 (0.59–1.45)	0.74	-	-
Duration on ART	1.21 (0.73–2.01)	0.46	-	-
PWV	1.01 (0.90–1.13)	0.86	**-**	**-**

*Unadjusted prevalence ratios (PR) for clinically relevant risk factors for non-dipping BP

**Variables with conservative p-value ≤0.25 retained in multivariable model to estimate adjusted PRs for factors associated with non-dipping BP

## Discussion

In this study of predominantly black females <50 years old on ART for more than 5 years, we found that, despite high rates of viral suppression, there was evidence of inflammation and vascular stiffness. A non-dipping pattern on 24hr ABPM was prevalent and could not be accounted for by established risk factors such as autonomic dysfunction, increased waist circumference and older age. These observations support the phenomenon of accelerated aging influenced by chronic inflammation in HIV infection.

De Luca *et al*. noted an 8-fold increase in cardiovascular risk in HIV-infected patients on ART with hsCRP levels >3.3 mg/L compared with those with levels <0.9 mg/L regardless of viral suppression.[11] A hsCRP >3.0mg/L was found in 69% of our cohort and was significantly associated with an increased waist circumference present in 80% of females. This would suggest an increase in cardiovascular risk in the majority of our cohort of young black females who traditionally are considered at a low risk for cardiovascular disease.

The prevalence of non-dipping BP was greater in our cohort than in a cohort of HIV-unknown, black Nigerian patients with more traditional risk factors for non-dipping BP (e.g. older age, more males) described by Isiguzo *et al*.[21] This suggests that non-traditional risk factors are contributory to the loss in diurnal BP variation in our black South African cohort. Our current prevalence of non-dipping BP was less than the 82% found in a smaller group of HIV-infected patients at baseline and after 6 months on ART.[22] The 82% non-dipping BP prevalence could possibly be explained by higher levels of inflammation expected with immune reconstitution (as this population was tested at ART initiation).[36] In addition, a larger percentage of the participants were male and smokers, both of which are risk factors for non-dipping BP.[18]

During a person’s lifetime, as part of the aging process or as a consequence of hypertension, atherosclerosis, or other pathological processes, the aorta stiffens. The aortic PWV and CASP reflect central arterial stiffness. Substantial differences in central BP exist across different ethnic groups that cannot be explained by differences in conventional risk factors for cardiovascular disease.[37] Consequently, the CASP reading in our cohort ideally should be compared to a healthy, HIV-uninfected, age-matched black population. Unfortunately, no normative values in black Africans are available. Whereas, when compared to the small ‘normal’ black cohort of Shiburi *et al*[35], our HIV infected population had higher PWV readings suggesting greater aortic stiffness.

Although this cross-sectional design does not allow inference regarding causality, none of the traditional risk factors such as autonomic dysfunction and obesity were found to be associated with non-dipping BP. This finding is consistent with a systematic review and meta-analysis by Kent *et al*. on ABPM profiles in HIV.[19] Thus, HIV infection itself or other non- traditional potential risk factors for non-dipping BP such as: mood disorders, psychosocial stress and poor sleep quality need to be considered. Health care providers should consequently be attentive towards diagnosing psychosocial stressors and mood disorders, depression in particular, which are the most frequent psychiatric complication associated with HIV.[38]

The present study must be interpreted within the context of its limitations. The study cohort is relatively small and cross-sectional and a matched HIV-uninfected control group was lacking. 91% of the cohort was female, similar to other South African HIV infected cohorts[24,25], possibly explained by the greater likelihood of African women to: seek and use health care, have greater knowledge about health, be compliant with their therapy and monitor the health of others as well as their own health.[39] Longitudinal observational data would be required to better determine causality for non-dipping BP in HIV populations. Although patients were asked about hours slept, their sleep quality and social stressors, which may be impacting on a loss of nocturnal BP dipping, were not explored. Due to the lack of accurate self- reporting the effect of the different ART regimens, on non-dipping BP, was not investigated.

## Conclusion

Our well, predominantly female and younger than middle age (<50 years), cohort of HIV-infected black South Africans had evidence of: obesity, vascular stiffness, non-dipping BP and high levels of inflammation. Cumulatively, these factors suggest an increased cardiovascular risk despite adequate viral suppression. These data need confirmation in larger samples and the impact on cardiovascular outcomes requires longitudinal studies. In the interim, it would be crucial to intensively screen for and address cardiovascular risk factors in HIV infected patients on ART.

## Supporting information

S1 File Data set. (PDF)

## References

[1] Deeks SG. HIV Infection, Inflammation, and Aging. 2011;20(October 2010). 10.1146/annurev-med-042909-093756 PMC3759035 21090961

[2] Wearne N, Okpechi IG. HIV-associated renal disease–an overview. Clin Nephrol [Internet]. 2016;86(S1):41–7. Available from: https://www.dustri.com/article_response_page.html?artId=14637&doi=10.5414/CNP86S117&L=0 10.5414/CNP86S117 27469157

[3] Zanni M V, Schouten J, Grinspoon SK, Reiss P. Risk of coronary heart disease in patients with HIV infection. Nat Rev Cardiol [Internet]. Nature Publishing Group, a division of Macmillan Publishers Limited. All Rights Reserved.; 2014 12;11(12):728–41. Available from: 10.1038/nrcardio.2014.167 25331088

[4] Bloomfield GS, Hogan JW, Keter A, Sang E, Carter EJ, Velazquez EJ, et al Hypertension and Obesity as Cardiovascular Risk Factors among HIV Seropositive Patients in Western Kenya. PLoS One [Internet]. Public Library of Science; 2011 7 14;6(7):e22288 Available from: http://dx.doi.org/10.1371%2Fjournal.pone.0022288 10.1371/journal.pone.0022288 PMC3136516 21779407

[5] Fourie CMT, Van Rooyen JM, Schutte AE. HIV infection and cardiovascular risk in black South Africans. Cardiovasc J Afr [Internet]. Clinics Cardive Publishing; 2011 6;22(3):117–9. Available from: http://www.ncbi.nlm.nih.gov/pmc/articles/PMC3734753/PMC3734753 21713298

[6] Nixon DE, Landay AL. Biomarkers of immune dysfunction in HIV. Curr Opin HIV AIDS [Internet]. 2010 11;5(6):498–503. Available from: http://www.ncbi.nlm.nih.gov/pmc/articles/PMC3032605/10.1097/COH.0b013e32833ed6f4 PMC3032605 20978393

[7] JR W, NG K. Immunological Profiles in HIV Positive Patients with or without Opportunistic Infections and the Influence of Highly Active Antiretroviral Therapy: A Systematic Review and Update. J Clin Cell Immunol. 2016;7(3).

[8] Vishwanath A, Quaiser S, Khan R. Role of high-sensitivity C-reactive protein measurements in HIV patients. Indian J Sex Transm Dis [Internet]. India: Medknow Publications & Media Pvt Ltd; 2016;37(2):123–8. Available from: http://www.ncbi.nlm.nih.gov/pmc/articles/PMC5111295/10.4103/2589-0557.192127 PMC5111295 27890944

[9] Ross AC, Rizk N, O’Riordan MA, Dogra V, El-Bejjani D, Storer N, et al Relationship between Inflammatory Markers, Endothelial Activation Markers, and Carotid Intima-Media Thickness in HIV-Infected Patients Receiving Antiretroviral Therapy. Clin Infect Dis [Internet]. 2009 10 1;49(7):1119–27. Available from: http://www.ncbi.nlm.nih.gov/pmc/articles/PMC3895473/10.1086/605578 PMC3895473 19712036

[10] Kuller LH, Tracy R, Belloso W, De Wit S, Drummond F, Lane HC, et al Inflammatory and Coagulation Biomarkers and Mortality in Patients with HIV Infection. Deeks S, editor. PLoS Med [Internet]. San Francisco, USA: Public Library of Science; 2008 10 21;5(10):e203 Available from: http://www.ncbi.nlm.nih.gov/pmc/articles/PMC2570418/10.1371/journal.pmed.0050203 PMC2570418 18942885

[11] De L a, de Gaetano DK, Colafigli M, Cozzi-Lepri A, De C a, Gori A, et al The association of high-sensitivity c-reactive protein and other biomarkers with cardiovascular disease in patients treated for HIV: a nested case-control study. BMCInfectDis. BMC Infectious Diseases; 2013;13(1471–2334 (Electronic)):414. 24004495 10.1186/1471-2334-13-414 PMC3846422

[12] Duprez DA, Neuhaus J, Kuller LH, Tracy R, Belloso W, De Wit S, et al Inflammation, Coagulation and Cardiovascular Disease in HIV-Infected Individuals. PLoS One [Internet]. Public Library of Science; 2012 9 10;7(9):e44454 Available from: http://dx.doi.org/10.1371%2Fjournal.pone.0044454 10.1371/journal.pone.0044454 PMC3438173 22970224

[13] Dolan E, Stanton A, Thijs L, Hinedi K, Atkins N, McClory S, et al Superiority of Ambulatory Over Clinic Blood Pressure Measurement in Predicting Mortality. Hypertension [Internet]. 2005 6 23;46(1):156 LP–161. Available from: http://hyper.ahajournals.org/content/46/1/156.abstract 10.1161/01.HYP.0000170138.56903.7a 15939805

[14] Kent ST, Bromfield SG, Burkholder GA, Falzon L, Oparil S, Overton ET, et al Ambulatory blood pressure monitoring in individuals with HIV: A systematic review and meta-analysis. PLoS One [Internet]. 2016;11(2):1–17. Available from: 10.1371/journal.pone.0148920 PMC4755611 26882469

[15] Lazar JM, Wu X, Shi Q, Kagame A, Cohen M, Binagwaho A, et al Arterial wave reflection in HIV-infected and HIV-uninfected Rwandan women. AIDS Res Hum Retroviruses [Internet]. 2009;25(9):877–82. Available from: http://www.pubmedcentral.nih.gov/articlerender.fcgi?artid=2858930&tool=pmcentrez&rendertype=abstract 10.1089/aid.2008.0269 PMC2858930 19689195

[16] Rider OJ, Asaad M, Ntusi N, Wainwright E, Clutton G, Hancock G, et al HIV is an independent predictor of aortic stiffness. J Cardiovasc Magn Reson [Internet]. 2014;16(1):57 Available from: http://www.biomedcentral.com/content/pdf/s12968-014-0057-1.pdf%5Cn http://www.ncbi.nlm.nih.gov/pubmed/25187084 10.1186/s12968-014-0057-1 PMC4422254 25187084

[17] Echeverría P, Bonjoch A, Moltó J, Jou A, Puig J, Ornelas A, et al Pulse Wave Velocity as Index of Arterial Stiffness in HIV-Infected Patients Compared With a Healthy Population. 2014;65(1):50–6. 10.1097/QAI.0b013e3182a97c17 23982659

[18] Profant J, Dimsdale JE. Race and diurnal blood pressure patterns. A review and meta-analysis. Hypertension. 1999;33(5):1099–104. 10334794 10.1161/01.hyp.33.5.1099

[19] Kent ST, Schwartz JE, Shimbo D, Overton ET, Burkholder GA, Oparil S, et al Race and Sex Differences in Ambulatory Blood Pressure Measures among HIV+ Adults. J Am Soc Hypertens [Internet]. Elsevier Inc.; 2017; Available from: http://linkinghub.elsevier.com/retrieve/pii/S193317111730150X 10.1016/j.jash.2017.05.002 PMC5667355 28624171

[20] Morar N, Seedat YK, Naidoo DP, Desai DK. Ambulatory blood pressure and risk factors for coronary heart disease in black and Indian medical students. J Cardiovasc Risk. England; 1998;5(5–6):313–8. 9920002

[21] Isiguzo G, Baugh D, Nwuruku G, Mezue K, Madu C, Madu E. Initial experience with 24-h ambulatory blood pressure monitoring in Nigerian patients with hypertension. Niger J Cardiol [Internet]. 2016;13(1):33–8. Available from: http://www.nigjcardiol.org/text.asp?2016/13/1/33/173851

[22] Borkum M, Alfred A, Wearne N, Rayner B, Dave JA, Levitt NS. Ambulatory blood pressure profiles in a subset of HIV-positive patients pre and post antiretroviral therapy. Cardiovasc J Afr [Internet]. Clinics Cardive Publishing; 2014 10 6;25(4):153–7. Available from: http://www.ncbi.nlm.nih.gov/pmc/articles/PMC4170173/10.5830/CVJA-2014-029 PMC4170173 25192297

[23] Dave JA, Levitt NS, Ross IL, Lacerda M, Maartens G, Blom D. Anti-Retroviral Therapy Increases the Prevalence of Dyslipidemia in South African HIV-Infected Patients. PLoS One [Internet]. Public Library of Science; 2016 1 17 [cited 2017 Jan 21];11(3):e0151911 Available from: 10.1371/journal.pone.0151911 PMC4795704 26986065

[24] Centner CM, Little F, Van Der Watt JJ, Vermaak JR, Dave JA, Levitt NS, Heckmann JM. Evolution of sensory neuropathy after initiation of antiretroviral therapy. Muscle and Nerve. United States; Muscle Nerve. 2017 5 31 10.1002/mus.25710 [Epub ahead of print] 28561925

[25] Maritz J, Benatar M, Dave JA, Harrison TB, Badri M, Levitt NS, et al HIV neuropathy in South Africans: frequency, characteristics, and risk factors. Muscle Nerve. United States; 2010 5;41(5):599–606. 10.1002/mus.21535 20229576

[26] Kilic H, Yelgec S, Salih O, Akdemir R, Karakurt O, Cagirci G, et al An invasive but simple and accurate method for ascending aorta–femoral artery pulse wave velocity measurement. Blood Press. Taylor & Francis; 2013;22(1):45–50. 10.3109/08037051.2012.694255 22747433

[27] Zilliox L, Peltier AC, Wren PA, Anderson A, Smith AG, Singleton JR, et al Assessing autonomic dysfunction in early diabetic neuropathy: The Survey of Autonomic Symptoms. Neurology. 2011;76(12):1099–105. 10.1212/WNL.0b013e3182120147 21422460 PMC3068012

[28] Stranieri A, Abawajy J, Kelarev A, Huda S, Chowdhury M, Jelinek HF. An approach for Ewing test selection to support the clinical assessment of cardiac autonomic neuropathy. Artif Intell Med. Netherlands; 2013 7;58(3):185–93. 10.1016/j.artmed.2013.04.007 23768975

[29] Yehia BR, Fleishman JA, Metlay JP, Moore RD, Gebo KA. Sustained Viral Suppression in HIV-Infected Patients Receiving Antiretroviral Therapy. JAMA. 2012 7 25;308(4):339–42. 10.1001/jama.2012.5927 22820781 PMC3541503

[30] Pearson TA, Mensah GA, Alexander RW, Anderson JL, Cannon RO, Criqui M, et al Markers of inflammation and cardiovascular disease: Application to clinical and public health practice: A statement for healthcare professionals from the centers for disease control and prevention and the American Heart Association. Circulation. 2003;107(3):499–511. 12551878 10.1161/01.cir.0000052939.59093.45

[31] Levey AS, Stevens LA, Schmid CH, Zhang YL, Castro AF 3rd, Feldman HI, et al A new equation to estimate glomerular filtration rate. Ann Intern Med. United States; 2009 5;150(9):604–12. 10.7326/0003-4819-150-9-200905050-00006 PMC2763564 19414839

[32] Seedat YK, Rayner BL, Veriava Y. South African hypertension practice guideline 2014. Cardiovasc J Afr [Internet]. Clinics Cardive Publishing; 2014 8 5;25(6):288–94. Available from: http://www.ncbi.nlm.nih.gov/pmc/articles/PMC4327181/10.5830/CVJA-2014-062 PMC4327181 25629715

[33] Alberti KGMM, Eckel RH, Grundy SM, Zimmet PZ, Cleeman JI, Donato KA, et al Harmonizing the Metabolic Syndrome: A Joint Interim Statement of the International Diabetes Federation Task Force on Epidemiology and Prevention; National Heart, Lung, and Blood Institute; American Heart Association; World Heart Federation; International. Circulation [Internet]. 2009;120(16):1640–5. Available from: http://circ.ahajournals.org/content/120/16/1640.abstract%5Cn http://circ.ahajournals.org/content/120/16/1640.full.pdf 10.1161/CIRCULATIONAHA.109.192644 19805654

[34] World Health Organization. De nition and diagnosis of diabetes mellitus and intermediate hyperglycaemia [homepage on the Internet]. c2006 [cited 2011 Sep 20].

[35] Shiburi CP, Staessen JA, Maseko M, Wojciechowska W, Thijs L, Van Bortel LM, et al Reference values for SphygmoCor measurements in South Africans of African ancestry. Am J Hypertens. 2006;19(1):40–6. 10.1016/j.amjhyper.2005.06.018 16461189

[36] Van Watt Der JJ, Wilkinson KA, Wilkinson RJ, Heckmann JM. Plasma cytokine profiles in HIV-1 infected patients developing neuropathic symptoms shortly after commencing antiretroviral therapy: a case-control study. BMC Infectious Diseases. 2014; 14:71 Available from: 10.1186/1471-2334-14-71 24512313 PMC3928502

[37] Eeftinck Schattenkerk DW, van Gorp J, Snijder MB, Zwinderman AH, Agyemang CO, Peters RJG, et al Ethnic Differences in Arterial Wave Reflection Are Mostly Explained by Differences in Body Height—Cross-Sectional Analysis of the HELIUS Study. PLoS One [Internet]. 2016;11(7):e0160243 Available from: http://dx.plos.org/10.1371/journal.pone.0160243 10.1371/journal.pone.0160243 PMC4966932 27472397

[38] Bhatia MS, Munjal S. Prevalence of Depression in People Living with HIV/AIDS Undergoing ART and Factors Associated with it. J Clin Diagn Res [Internet]. Delhi, India: JCDR Research and Publications (P) Limited; 2014 10 20;8(10):WC01–WC04. Available from: http://www.ncbi.nlm.nih.gov/pmc/articles/PMC4253251/10.7860/JCDR/2014/7725.4927 PMC4253251 25478433

[39] van der Hoeven M, Kruger A, Greeff M. Differences in health care seeking behaviour between rural and urban communities in South Africa. Int J Equity Health [Internet]. BioMed Central; 2012 6 12;11:31 Available from: http://www.ncbi.nlm.nih.gov/pmc/articles/PMC3419677/10.1186/1475-9276-11-31 PMC3419677 22691443

