# Supplementary material for: High prevalence of “non-dipping” blood pressure and vascular stiffness in HIV-infected South Africans on antiretrovirals
Source: PLoS One. 2017 Sep 20;12(9):e0185003. doi: 10.1371/journal.pone.0185003 (PMC5607221; doi:10.1371/journal.pone.0185003)
Supplement: S1 File — (PDF) [file pone.0185003.s001.pdf]

```
------------------------------------------------------------------------------------------------------------------------
-------------------------------
      name:  <unnamed>
       log:  /Users/grethenothling/Box Sync/Stats Sync/Megan B/Output/March 2017/Megan B cross-sectional analysis 
10_03_2017.log
  log type:  text
 opened on:  10 Mar 2017, 15:56:29

. do "/var/folders/bc/s5r3kgnd26d2m5s1fcv0knhw0000gp/T//SD24734.000000"

. ********************************************************************************
. *************************   MEGAN B final analysis  ***************************
. ********************************************************************************
. 
. 
. ********************************************************************************
. *************************            General & Socio-demographics       *********
. ********************************************************************************
. mdesc

    Variable    |     Missing          Total     Percent Missing
----------------+-----------------------------------------------
             no |           0             67           0.00
       visitkey |           0             67           0.00
    testingdate |           0             67           0.00
            dob |           0             67           0.00
            age |           0             67           0.00
        agecat3 |           0             67           0.00
        agecat2 |           0             67           0.00
         agecat |           0             67           0.00
     previoustb |           0             67           0.00
         whentb |           6             67           8.96
      currenttb |           1             67           1.49
        art_d4t |           1             67           1.49
        art_azt |           1             67           1.49
        art_3tc |           1             67           1.49
        art_efv |           1             67           1.49
        art_nvp |           1             67           1.49
        art_tdf |           1             67           1.49
        art_ftc |           1             67           1.49
     lpvrit_alu |           1             67           1.49
     failedreg1 |           1             67           1.49
   lastarvcha~e |           2             67           2.99
         vitbco |           2             67           2.99
          vitb6 |           2             67           2.99
        bactrim |           2             67           2.99
       cd4count |           7             67          10.45
    viralload_5 |           2             67           2.99
     viralsuppr |           3             67           4.48
            alt |          67             67         100.00
          creat |           1             67           1.49
            gfr |           1             67           1.49
           gfr2 |           1             67           1.49
         weight |           0             67           0.00
            bmi |           0             67           0.00
        bmicat2 |           0             67           0.00
         bmicat |           0             67           0.00
         height |           0             67           0.00
          waist |           1             67           1.49
   waistcat_f~2 |           6             67           8.96
   waistcat_m~2 |          61             67          91.04
         cd4cat |          67             67         100.00
       cd4catbl |          67             67         100.00
   alcohol_ever |           0             67           0.00
   alc_last_y~r |           0             67           0.00
          fbg_0 |           1             67           1.49
          g_120 |           1             67           1.49
     ogtt_class |          14             67          20.90
         homair |          67             67         100.00
             tc |           1             67           1.49
             tg |           1             67           1.49
            hdl |           1             67           1.49
            ldl |           1             67           1.49
          sbp_1 |           0             67           0.00
          dbp_1 |           0             67           0.00
     uric_acid  |           5             67           7.46
         hs_crp |           1             67           1.49
      hscrp_cat |           1             67           1.49
     microalb_1 |           0             67           0.00
     microalb_2 |           3             67           4.48
   microalb_cat |           1             67           1.49
       pos_drop |           1             67           1.49
    pos_symptom |           1             67           1.49
      rr_abnorm |           1             67           1.49
         cap_30 |          60             67          89.55
      cap_30_39 |          45             67          67.16
      cap_40_49 |          44             67          65.67
   cap_40_49_~e |          64             67          95.52
      cap_50_59 |          60             67          89.55
   cap_50_59_~e |          64             67          95.52
         cap_60 |          65             67          97.01
```

```
             cap |          0           67          0.00
         cap_low |          0           67          0.00
        cap_high |          0           67          0.00
           pp_30 |         60           67         89.55
        ppcat_30 |         60           67         89.55
        pp_30_49 |         19           67         28.36
     ppcat_30_49 |         19           67         28.36
           pp_50 |         55           67         82.09
        ppcat_50 |         55           67         82.09
              pp |          0           67          0.00
           ppcat |          0           67          0.00
          pp_low |          0           67          0.00
         pp_high |          0           67          0.00
              ap |          0           67          0.00
          ap_low |          0           67          0.00
         ap_high |          0           67          0.00
          aix_30 |         60           67         89.55
       aixcat_30 |         60           67         89.55
       aix_30_49 |         19           67         28.36
    aixcat_30_49 |         19           67         28.36
          aix_50 |         55           67         82.09
       aixcat_50 |         55           67         82.09
             aix |          0           67          0.00
          aixcat |          0           67          0.00
         aix_low |          0           67          0.00
        aix_high |          0           67          0.00
             pwv |          2           67          2.99
         sbp_day |          1           67          1.49
       sbp_night |          1           67          1.49
         dbp_day |          1           67          1.49
       dbp_night |          1           67          1.49
             dip |          1           67          1.49
       sbpchange |          1           67          1.49
    sbpchange_~t |          1           67          1.49
          hr_day |          1           67          1.49
        hr_night |          1           67          1.49
     reverse_dip |          0           67          0.00
         sbp_140 |          0           67          0.00
          dbp_90 |          0           67          0.00
             hpt |          0           67          0.00
        mets_SBP |          0           67          0.00
        mets_DBP |          0           67          0.00
          sex_m1 |          0           67          0.00
     mets_femHDL |          6           67          8.96
    mets_maleHDL |         61           67         91.04
         metS_WC |          0           67          0.00
         metS_TG |          0           67          0.00
        metS_HDL |          0           67          0.00
         metS_BP |          0           67          0.00
       metS_gluc |          0           67          0.00
        metS_tot |          0           67          0.00
----------------+---------------------------------------------

.
. univar age
                                        -------------- Quantiles --------------
Variable       n     Mean     S.D.      Min      .25      Mdn      .75      Max
-------------------------------------------------------------------------------
     age      67    42.16     8.59    26.44    36.00    41.39    46.74    66.41
-------------------------------------------------------------------------------

.
.
. tab sex

    Sex_M-1 |      Freq.     Percent        Cum.
------------+-----------------------------------
     Female |         61       91.04       91.04
       Male |          6        8.96      100.00
------------+-----------------------------------
      Total |         67      100.00

.
.
. univar weight, by (sex)

-> sex_m1=Female
                                        -------------- Quantiles --------------
Variable       n     Mean     S.D.      Min      .25      Mdn      .75      Max
-------------------------------------------------------------------------------
  weight      61    72.20    16.66    42.00    62.00    71.00    82.70   117.00
-------------------------------------------------------------------------------

-> sex_m1=Male
                                        -------------- Quantiles --------------
Variable       n     Mean     S.D.      Min      .25      Mdn      .75      Max
-------------------------------------------------------------------------------
  weight       6    62.62     8.75    51.00    56.20    62.50    69.00    74.50
-------------------------------------------------------------------------------

.
```

```
.
. univar bmi, by (sex)

-> sex_m1=Female
                                        -------------- Quantiles --------------
Variable       n     Mean     S.D.      Min      .25      Mdn      .75      Max
-------------------------------------------------------------------------------
     bmi      61    27.56     8.13     0.00    23.27    27.69    32.05    46.62
-------------------------------------------------------------------------------

-> sex_m1=Male
                                        -------------- Quantiles --------------
Variable       n     Mean     S.D.      Min      .25      Mdn      .75      Max
-------------------------------------------------------------------------------
     bmi       6    22.57     2.58    19.68    20.64    21.91    24.88    26.40
-------------------------------------------------------------------------------

. *** patient 286 is only underweight patient BMI 14.5
.
. tab bmicat2

      bmicat2 |      Freq.     Percent        Cum.
--------------+-----------------------------------
  Underweight |          3        4.48        4.48
Normal weight |         20       29.85       34.33
   Overweight |         22       32.84       67.16
        Obese |         22       32.84      100.00
--------------+-----------------------------------
        Total |         67      100.00

.
. tab bmicat2 sex, col

+-------------------+
| Key               |
|-------------------|
|     frequency     |
| column percentage |
+-------------------+

              |       Sex_M-1
      bmicat2 |    Female       Male |     Total
--------------+----------------------+----------
  Underweight |         3          0 |         3
              |      4.92       0.00 |      4.48
--------------+----------------------+----------
Normal weight |        15          5 |        20
              |     24.59      83.33 |     29.85
--------------+----------------------+----------
   Overweight |        21          1 |        22
              |     34.43      16.67 |     32.84
--------------+----------------------+----------
        Obese |        22          0 |        22
              |     36.07       0.00 |     32.84
--------------+----------------------+----------
        Total |        61          6 |        67
              |    100.00     100.00 |    100.00


.
. univar waist, by (sex)

-> sex_m1=Female
                                        -------------- Quantiles --------------
Variable       n     Mean     S.D.      Min      .25      Mdn      .75      Max
-------------------------------------------------------------------------------
   waist      60    92.28    14.73    62.50    83.00    91.00   100.75   126.00
-------------------------------------------------------------------------------

-> sex_m1=Male
                                        -------------- Quantiles --------------
Variable       n     Mean     S.D.      Min      .25      Mdn      .75      Max
-------------------------------------------------------------------------------
   waist       6    87.67    15.25    72.00    80.00    81.00    99.00   113.00
-------------------------------------------------------------------------------

.
. tab waistcat_fem2

waistcat_fe |
         m2 |      Freq.     Percent        Cum.
------------+-----------------------------------
      <80cm |         12       19.67       19.67
      >80cm |         49       80.33      100.00
------------+-----------------------------------
      Total |         61      100.00

.
.
. tab waistcat_male2
```

```
waistcat_ma |
        le2 |      Freq.     Percent        Cum.
------------+-----------------------------------
      <94cm |          4       66.67       66.67
      >94cm |          2       33.33      100.00
------------+-----------------------------------
      Total |          6      100.00

.
.
. univar cd4count
                                        -------------- Quantiles --------------
Variable       n     Mean     S.D.      Min      .25      Mdn      .75      Max
-------------------------------------------------------------------------------
cd4count      60   560.22   236.49    62.00   372.00   529.50   686.50  1318.00
-------------------------------------------------------------------------------

.
.
. tab viralsuppr

 ViralSuppr |      Freq.     Percent        Cum.
------------+-----------------------------------
          0 |         10       15.62       15.62
          1 |         54       84.38      100.00
------------+-----------------------------------
      Total |         64      100.00

.
. univar tc
                                        -------------- Quantiles --------------
Variable       n     Mean     S.D.      Min      .25      Mdn      .75      Max
-------------------------------------------------------------------------------
      tc      66     4.52     1.03     2.72     3.76     4.52     5.00     7.37
-------------------------------------------------------------------------------

. univar tg
                                        -------------- Quantiles --------------
Variable       n     Mean     S.D.      Min      .25      Mdn      .75      Max
-------------------------------------------------------------------------------
      tg      66     1.01     0.42     0.46     0.73     0.92     1.16     2.45
-------------------------------------------------------------------------------

. univar hdl
                                        -------------- Quantiles --------------
Variable       n     Mean     S.D.      Min      .25      Mdn      .75      Max
-------------------------------------------------------------------------------
     hdl      66     1.57     0.55     0.40     1.20     1.50     1.93     3.71
-------------------------------------------------------------------------------

. univar ldl
                                        -------------- Quantiles --------------
Variable       n     Mean     S.D.      Min      .25      Mdn      .75      Max
-------------------------------------------------------------------------------
     ldl      66     2.50     0.81     0.75     1.90     2.41     2.90     4.96
-------------------------------------------------------------------------------

.
.
. univar hs_crp
                                        -------------- Quantiles --------------
Variable       n     Mean     S.D.      Min      .25      Mdn      .75      Max
-------------------------------------------------------------------------------
  hs_crp      66    14.50    47.70     0.20     2.20     4.50     7.80   359.80
-------------------------------------------------------------------------------

.
. tab hscrp_cat

  hscrp_cat |      Freq.     Percent        Cum.
------------+-----------------------------------
         <1 |          8       12.12       12.12
        1-3 |         13       19.70       31.82
         >3 |         45       68.18      100.00
------------+-----------------------------------
      Total |         66      100.00

.
. univar uric_acid
                                        -------------- Quantiles --------------
Variable       n     Mean     S.D.      Min      .25      Mdn      .75      Max
-------------------------------------------------------------------------------
uric_acid      62     0.25     0.09     0.08     0.18     0.23     0.30     0.51
-------------------------------------------------------------------------------

.
.
.
. univar sbp_day
                                        -------------- Quantiles --------------
Variable       n     Mean     S.D.      Min      .25      Mdn      .75      Max
```

```
--------------------------------------------------------------------------------
 sbp_day      66   119.61    17.40    90.00   107.00   118.00   127.00   175.00
--------------------------------------------------------------------------------

.
. univar dbp_day
                                        -------------- Quantiles --------------
Variable       n     Mean     S.D.      Min      .25      Mdn      .75      Max
--------------------------------------------------------------------------------
 dbp_day      66    78.39    12.65    58.00    69.00    76.50    85.00   126.00
--------------------------------------------------------------------------------

.
. univar sbp_night
                                        -------------- Quantiles --------------
Variable       n     Mean     S.D.      Min      .25      Mdn      .75      Max
--------------------------------------------------------------------------------
sbp_night      66   109.80    19.17    73.00    96.00   108.00   117.00   173.00
--------------------------------------------------------------------------------

.
. univar dbp_night
                                        -------------- Quantiles --------------
Variable       n     Mean     S.D.      Min      .25      Mdn      .75      Max
--------------------------------------------------------------------------------
dbp_night      66    67.82    13.67    50.00    58.00    65.00    77.00   113.00
--------------------------------------------------------------------------------

.
.
.
. tab ogtt_class

 OGTT_class |      Freq.     Percent        Cum.
------------+-----------------------------------
          0 |         49       92.45       92.45
          1 |          2        3.77       96.23
          2 |          2        3.77      100.00
------------+-----------------------------------
      Total |         53      100.00

.
.
. tab  metS_tot

   metS_tot |      Freq.     Percent        Cum.
------------+-----------------------------------
          0 |          7       10.45       10.45
          1 |         21       31.34       41.79
          2 |         26       38.81       80.60
          3 |         10       14.93       95.52
          4 |          3        4.48      100.00
------------+-----------------------------------
      Total |         67      100.00

.
.
. univar creat
                                        -------------- Quantiles --------------
Variable       n     Mean     S.D.      Min      .25      Mdn      .75      Max
--------------------------------------------------------------------------------
   creat      66    63.86    12.10    43.00    56.00    62.00    69.00   111.00
--------------------------------------------------------------------------------

.
. univar gfr2
                                        -------------- Quantiles --------------
Variable       n     Mean     S.D.      Min      .25      Mdn      .75      Max
--------------------------------------------------------------------------------
    gfr2      66    96.87    18.56    48.60    89.99    90.45   106.00   157.40
--------------------------------------------------------------------------------

.
.
. tab dip

        dip |      Freq.     Percent        Cum.
------------+-----------------------------------
     dipper |         23       34.85       34.85
 non-dipper |         43       65.15      100.00
------------+-----------------------------------
      Total |         66      100.00

.
. ********************************************************************************
. *************************            TABLE 2        ***************************
. ********************************************************************************
.
. *************************      Dip comparisons      ***************************
.
.
```

```
.
. tab dip

        dip |      Freq.     Percent        Cum.
------------+-----------------------------------
     dipper |         23       34.85       34.85
 non-dipper |         43       65.15      100.00
------------+-----------------------------------
      Total |         66      100.00

.
. univar age, by (dip)

-> dip=dipper
                                        -------------- Quantiles --------------
Variable       n     Mean     S.D.      Min      .25      Mdn      .75      Max
-------------------------------------------------------------------------------
     age      23    43.04     8.85    26.44    35.60    45.05    51.07    57.95
-------------------------------------------------------------------------------

-> dip=non-dipper
                                        -------------- Quantiles --------------
Variable       n     Mean     S.D.      Min      .25      Mdn      .75      Max
-------------------------------------------------------------------------------
     age      43    41.83     8.56    27.95    36.02    40.88    46.44    66.41
-------------------------------------------------------------------------------

-> dip=.
                                        -------------- Quantiles --------------
Variable       n     Mean     S.D.      Min      .25      Mdn      .75      Max
-------------------------------------------------------------------------------
     age       1    35.56        .    35.56    35.56    35.56    35.56    35.56
-------------------------------------------------------------------------------

. ranksum age, by (dip)

Two-sample Wilcoxon rank-sum (Mann-Whitney) test

         dip |      obs    rank sum    expected
-------------+---------------------------------
      dipper |       23         821       770.5
  non-dipper |       43        1390      1440.5
-------------+---------------------------------
    combined |       66        2211        2211

unadjusted variance     5521.92
adjustment for ties        0.00
                     ----------
adjusted variance       5521.92

Ho: age(dip==dipper) = age(dip==non-dipper)
             z =   0.680
    Prob > |z| =   0.4968

.
. tab sex_m1 dip, col exact

+-------------------+
| Key               |
|-------------------|
|     frequency     |
| column percentage |
+-------------------+

           |          dip
   Sex_M-1 |    dipper  non-dippe |     Total
-----------+----------------------+----------
    Female |        19         41 |        60
           |     82.61      95.35 |     90.91
-----------+----------------------+----------
      Male |         4          2 |         6
           |     17.39       4.65 |      9.09
-----------+----------------------+----------
     Total |        23         43 |        66
           |    100.00     100.00 |    100.00

           Fisher's exact =                 0.172
   1-sided Fisher's exact =                 0.105

.
.
. univar sbp_1, by (dip)

-> dip=dipper
                                        -------------- Quantiles --------------
Variable       n     Mean     S.D.      Min      .25      Mdn      .75      Max
-------------------------------------------------------------------------------
   sbp_1      23   119.87    23.27    89.00   106.00   113.00   133.00   187.00
-------------------------------------------------------------------------------

-> dip=non-dipper
```

```
                                        -------------- Quantiles --------------
Variable       n     Mean     S.D.      Min      .25      Mdn      .75      Max
-------------------------------------------------------------------------------
   sbp_1      43   127.77    18.12   100.00   116.00   127.00   137.00   184.00
-------------------------------------------------------------------------------

-> dip=.
                                        -------------- Quantiles --------------
Variable       n     Mean     S.D.      Min      .25      Mdn      .75      Max
-------------------------------------------------------------------------------
   sbp_1       1   140.00        .   140.00   140.00   140.00   140.00   140.00
-------------------------------------------------------------------------------

. ranksum sbp_1, by (dip)

Two-sample Wilcoxon rank-sum (Mann-Whitney) test

         dip |      obs    rank sum    expected
-------------+---------------------------------
      dipper |       23         662       770.5
  non-dipper |       43        1549      1440.5
-------------+---------------------------------
    combined |       66        2211        2211

unadjusted variance     5521.92
adjustment for ties       -6.80
                      ----------
adjusted variance       5515.12

Ho: sbp_1(dip==dipper) = sbp_1(dip==non-dipper)
             z =  -1.461
    Prob > |z| =   0.1440

.
.
. univar dbp_1, by (dip)

-> dip=dipper
                                        -------------- Quantiles --------------
Variable       n     Mean     S.D.      Min      .25      Mdn      .75      Max
-------------------------------------------------------------------------------
   dbp_1      23    75.52    15.69    55.00    63.00    74.00    84.00   124.00
-------------------------------------------------------------------------------

-> dip=non-dipper
                                        -------------- Quantiles --------------
Variable       n     Mean     S.D.      Min      .25      Mdn      .75      Max
-------------------------------------------------------------------------------
   dbp_1      43    79.37    13.56    57.00    69.00    78.00    93.00   111.00
-------------------------------------------------------------------------------

-> dip=.
                                        -------------- Quantiles --------------
Variable       n     Mean     S.D.      Min      .25      Mdn      .75      Max
-------------------------------------------------------------------------------
   dbp_1       1    94.00        .    94.00    94.00    94.00    94.00    94.00
-------------------------------------------------------------------------------

. ranksum dbp_1, by (dip)

Two-sample Wilcoxon rank-sum (Mann-Whitney) test

         dip |      obs    rank sum    expected
-------------+---------------------------------
      dipper |       23         677       770.5
  non-dipper |       43        1534      1440.5
-------------+---------------------------------
    combined |       66        2211        2211

unadjusted variance     5521.92
adjustment for ties       -6.92
                      ----------
adjusted variance       5515.00

Ho: dbp_1(dip==dipper) = dbp_1(dip==non-dipper)
             z =  -1.259
    Prob > |z| =   0.2080

.
. univar bmi, by (dip)

-> dip=dipper
                                        -------------- Quantiles --------------
Variable       n     Mean     S.D.      Min      .25      Mdn      .75      Max
-------------------------------------------------------------------------------
     bmi      23    25.81     5.55    14.53    21.55    25.97    30.49    35.85
-------------------------------------------------------------------------------

-> dip=non-dipper
                                        -------------- Quantiles --------------
Variable       n     Mean     S.D.      Min      .25      Mdn      .75      Max
```

```
------------------------------------------------------------------------------
     bmi      43    27.55     8.84     0.00    23.27    27.93    32.05    46.62
------------------------------------------------------------------------------

-> dip=.
                                        -------------- Quantiles --------------
Variable       n     Mean     S.D.      Min      .25      Mdn      .75      Max
------------------------------------------------------------------------------
     bmi       1    38.20        .    38.20    38.20    38.20    38.20    38.20
------------------------------------------------------------------------------

. ranksum bmi, by (dip)

Two-sample Wilcoxon rank-sum (Mann-Whitney) test

         dip |      obs    rank sum    expected
-------------+---------------------------------
      dipper |       23         668       770.5
  non-dipper |       43        1543      1440.5
-------------+---------------------------------
    combined |       66        2211        2211

unadjusted variance     5521.92
adjustment for ties        0.00
                     ----------
adjusted variance       5521.92

Ho: bmi(dip==dipper) = bmi(dip==non-dipper)
             z =  -1.379
    Prob > |z| =   0.1678

.
. tab bmicat dip, col exact

+-------------------+
| Key               |
|-------------------|
|     frequency     |
| column percentage |
+-------------------+

Enumerating sample-space combinations:
stage 3:  enumerations = 1
stage 2:  enumerations = 4
stage 1:  enumerations = 0

              |          dip
       bmicat |    dipper  non-dippe |     Total
--------------+----------------------+----------
Normal weight |        10         13 |        23 
              |     43.48      30.23 |     34.85 
--------------+----------------------+----------
   Overweight |         7         15 |        22 
              |     30.43      34.88 |     33.33 
--------------+----------------------+----------
        Obese |         6         15 |        21 
              |     26.09      34.88 |     31.82 
--------------+----------------------+----------
        Total |        23         43 |        66 
              |    100.00     100.00 |    100.00 

           Fisher's exact =                 0.586

.
. tab ogtt_class dip, col exact

+-------------------+
| Key               |
|-------------------|
|     frequency     |
| column percentage |
+-------------------+

Enumerating sample-space combinations:
stage 3:  enumerations = 1
stage 2:  enumerations = 1
stage 1:  enumerations = 0

           |          dip
OGTT_class |    dipper  non-dippe |     Total
-----------+----------------------+----------
         0 |        19         30 |        49 
           |     95.00      90.91 |     92.45 
-----------+----------------------+----------
         1 |         0          2 |         2 
           |      0.00       6.06 |      3.77 
-----------+----------------------+----------
         2 |         1          1 |         2 
           |      5.00       3.03 |      3.77 
-----------+----------------------+----------
     Total |        20         33 |        53 
```

```
           |    100.00     100.00 |    100.00

           Fisher's exact =                 0.772

.
.
.
. univar fbg_0, by (dip)

-> dip=dipper
                                        -------------- Quantiles --------------
Variable       n     Mean     S.D.      Min      .25      Mdn      .75      Max
-------------------------------------------------------------------------------
   fbg_0      23     5.04     1.13     3.90     4.40     4.90     5.30     9.60
-------------------------------------------------------------------------------

-> dip=non-dipper
                                        -------------- Quantiles --------------
Variable       n     Mean     S.D.      Min      .25      Mdn      .75      Max
-------------------------------------------------------------------------------
   fbg_0      43     5.28     2.46     2.70     4.40     4.90     5.30    20.40
-------------------------------------------------------------------------------

-> dip=.
                                        -------------- Quantiles --------------
Variable       n     Mean     S.D.      Min      .25      Mdn      .75      Max
-------------------------------------------------------------------------------
   fbg_0       0        .        .        .        .        .        .        .
-------------------------------------------------------------------------------

. ranksum fbg_0, by (dip)

Two-sample Wilcoxon rank-sum (Mann-Whitney) test

         dip |      obs    rank sum    expected
-------------+---------------------------------
      dipper |       23         733       770.5
  non-dipper |       43        1478      1440.5
-------------+---------------------------------
    combined |       66        2211        2211

unadjusted variance     5521.92
adjustment for ties      -24.21
                     ----------
adjusted variance       5497.71

Ho: fbg_0(dip==dipper) = fbg_0(dip==non-dipper)
             z =  -0.506
    Prob > |z| =   0.6130

.
. univar g_120, by (dip)

-> dip=dipper
                                        -------------- Quantiles --------------
Variable       n     Mean     S.D.      Min      .25      Mdn      .75      Max
-------------------------------------------------------------------------------
   g_120      23     5.90     2.57     3.40     4.70     5.00     6.80    16.20
-------------------------------------------------------------------------------

-> dip=non-dipper
                                        -------------- Quantiles --------------
Variable       n     Mean     S.D.      Min      .25      Mdn      .75      Max
-------------------------------------------------------------------------------
   g_120      43     6.07     3.24     3.40     4.30     5.50     7.00    24.10
-------------------------------------------------------------------------------

-> dip=.
                                        -------------- Quantiles --------------
Variable       n     Mean     S.D.      Min      .25      Mdn      .75      Max
-------------------------------------------------------------------------------
   g_120       0        .        .        .        .        .        .        .
-------------------------------------------------------------------------------

. ranksum g_120, by (dip)

Two-sample Wilcoxon rank-sum (Mann-Whitney) test

         dip |      obs    rank sum    expected
-------------+---------------------------------
      dipper |       23         755       770.5
  non-dipper |       43        1456      1440.5
-------------+---------------------------------
    combined |       66        2211        2211

unadjusted variance     5521.92
adjustment for ties       -6.92
                     ----------
adjusted variance       5515.00

Ho: g_120(dip==dipper) = g_120(dip==non-dipper)
```

```
             z =  -0.209
    Prob > |z| =   0.8347

.
.
. univar creat, by (dip)

-> dip=dipper
                                        -------------- Quantiles --------------
Variable       n     Mean     S.D.      Min      .25      Mdn      .75      Max
-------------------------------------------------------------------------------
   creat      23    66.35    15.81    48.00    55.00    63.00    72.00   111.00
-------------------------------------------------------------------------------

-> dip=non-dipper
                                        -------------- Quantiles --------------
Variable       n     Mean     S.D.      Min      .25      Mdn      .75      Max
-------------------------------------------------------------------------------
   creat      43    62.53     9.52    43.00    56.00    62.00    67.00    95.00
-------------------------------------------------------------------------------

-> dip=.
                                        -------------- Quantiles --------------
Variable       n     Mean     S.D.      Min      .25      Mdn      .75      Max
-------------------------------------------------------------------------------
   creat       0        .        .        .        .        .        .        .
-------------------------------------------------------------------------------

. ranksum creat, by (dip)

Two-sample Wilcoxon rank-sum (Mann-Whitney) test

         dip |      obs    rank sum    expected
-------------+---------------------------------
      dipper |       23       821.5       770.5
  non-dipper |       43      1389.5      1440.5
-------------+---------------------------------
    combined |       66        2211        2211

unadjusted variance     5521.92
adjustment for ties      -19.25
                     ----------
adjusted variance       5502.67

Ho: creat(dip==dipper) = creat(dip==non-dipper)
             z =   0.688
    Prob > |z| =   0.4918

.
.
. univar ldl, by (dip)

-> dip=dipper
                                        -------------- Quantiles --------------
Variable       n     Mean     S.D.      Min      .25      Mdn      .75      Max
-------------------------------------------------------------------------------
     ldl      23     2.24     0.52     1.08     1.80     2.20     2.79     3.13
-------------------------------------------------------------------------------

-> dip=non-dipper
                                        -------------- Quantiles --------------
Variable       n     Mean     S.D.      Min      .25      Mdn      .75      Max
-------------------------------------------------------------------------------
     ldl      43     2.63     0.90     0.75     2.00     2.60     3.08     4.96
-------------------------------------------------------------------------------

-> dip=.
                                        -------------- Quantiles --------------
Variable       n     Mean     S.D.      Min      .25      Mdn      .75      Max
-------------------------------------------------------------------------------
     ldl       0        .        .        .        .        .        .        .
-------------------------------------------------------------------------------

. ranksum ldl, by (dip)

Two-sample Wilcoxon rank-sum (Mann-Whitney) test

         dip |      obs    rank sum    expected
-------------+---------------------------------
      dipper |       23         647       770.5
  non-dipper |       43        1564      1440.5
-------------+---------------------------------
    combined |       66        2211        2211

unadjusted variance     5521.92
adjustment for ties       -2.54
                     ----------
adjusted variance       5519.38

Ho: ldl(dip==dipper) = ldl(dip==non-dipper)
             z =  -1.662
```

```
    Prob > |z| =   0.0964

.
. univar tg, by (dip)

-> dip=dipper
                                        -------------- Quantiles --------------
Variable       n     Mean     S.D.      Min      .25      Mdn      .75      Max
-------------------------------------------------------------------------------
      tg      23     1.05     0.42     0.53     0.73     0.94     1.25     1.93
-------------------------------------------------------------------------------

-> dip=non-dipper
                                        -------------- Quantiles --------------
Variable       n     Mean     S.D.      Min      .25      Mdn      .75      Max
-------------------------------------------------------------------------------
      tg      43     0.98     0.43     0.46     0.72     0.88     1.08     2.45
-------------------------------------------------------------------------------

-> dip=.
                                        -------------- Quantiles --------------
Variable       n     Mean     S.D.      Min      .25      Mdn      .75      Max
-------------------------------------------------------------------------------
      tg       0        .        .        .        .        .        .        .
-------------------------------------------------------------------------------

. ranksum tg, by (dip)

Two-sample Wilcoxon rank-sum (Mann-Whitney) test

         dip |      obs    rank sum    expected
-------------+---------------------------------
      dipper |       23       830.5       770.5
  non-dipper |       43      1380.5      1440.5
-------------+---------------------------------
    combined |       66        2211        2211

unadjusted variance     5521.92
adjustment for ties       -2.54
                     ----------
adjusted variance       5519.38

Ho: tg(dip==dipper) = tg(dip==non-dipper)
             z =   0.808
    Prob > |z| =   0.4193

.
.
. univar hdl, by (dip)

-> dip=dipper
                                        -------------- Quantiles --------------
Variable       n     Mean     S.D.      Min      .25      Mdn      .75      Max
-------------------------------------------------------------------------------
     hdl      23     1.56     0.72     0.40     1.08     1.36     1.91     3.71
-------------------------------------------------------------------------------

-> dip=non-dipper
                                        -------------- Quantiles --------------
Variable       n     Mean     S.D.      Min      .25      Mdn      .75      Max
-------------------------------------------------------------------------------
     hdl      43     1.58     0.45     0.76     1.27     1.52     1.97     2.63
-------------------------------------------------------------------------------

-> dip=.
                                        -------------- Quantiles --------------
Variable       n     Mean     S.D.      Min      .25      Mdn      .75      Max
-------------------------------------------------------------------------------
     hdl       0        .        .        .        .        .        .        .
-------------------------------------------------------------------------------

. ranksum hdl, by (dip)

Two-sample Wilcoxon rank-sum (Mann-Whitney) test

         dip |      obs    rank sum    expected
-------------+---------------------------------
      dipper |       23         707       770.5
  non-dipper |       43        1504      1440.5
-------------+---------------------------------
    combined |       66        2211        2211

unadjusted variance     5521.92
adjustment for ties       -1.61
                     ----------
adjusted variance       5520.30

Ho: hdl(dip==dipper) = hdl(dip==non-dipper)
             z =  -0.855
    Prob > |z| =   0.3927
```

```
.
.
. univar sbp_day, by (dip)

-> dip=dipper
                                        -------------- Quantiles --------------
Variable       n     Mean     S.D.      Min      .25      Mdn      .75      Max
-------------------------------------------------------------------------------
 sbp_day      23   118.70    15.35    94.00   106.00   118.00   132.00   152.00
-------------------------------------------------------------------------------

-> dip=non-dipper
                                        -------------- Quantiles --------------
Variable       n     Mean     S.D.      Min      .25      Mdn      .75      Max
-------------------------------------------------------------------------------
 sbp_day      43   120.09    18.56    90.00   107.00   118.00   127.00   175.00
-------------------------------------------------------------------------------

-> dip=.
                                        -------------- Quantiles --------------
Variable       n     Mean     S.D.      Min      .25      Mdn      .75      Max
-------------------------------------------------------------------------------
 sbp_day       0        .        .        .        .        .        .        .
-------------------------------------------------------------------------------

. ranksum sbp_day, by (dip)

Two-sample Wilcoxon rank-sum (Mann-Whitney) test

         dip |      obs    rank sum    expected
-------------+---------------------------------
      dipper |       23       759.5       770.5
  non-dipper |       43      1451.5      1440.5
-------------+---------------------------------
    combined |       66        2211        2211

unadjusted variance     5521.92
adjustment for ties       -4.96
                     ----------
adjusted variance       5516.96

Ho: sbp_day(dip==dipper) = sbp_day(dip==non-dipper)
             z =  -0.148
    Prob > |z| =   0.8823

.
.
. univar dbp_day, by (dip)

-> dip=dipper
                                        -------------- Quantiles --------------
Variable       n     Mean     S.D.      Min      .25      Mdn      .75      Max
-------------------------------------------------------------------------------
 dbp_day      23    77.30     9.37    62.00    70.00    77.00    83.00   102.00
-------------------------------------------------------------------------------

-> dip=non-dipper
                                        -------------- Quantiles --------------
Variable       n     Mean     S.D.      Min      .25      Mdn      .75      Max
-------------------------------------------------------------------------------
 dbp_day      43    78.98    14.16    58.00    69.00    75.00    89.00   126.00
-------------------------------------------------------------------------------

-> dip=.
                                        -------------- Quantiles --------------
Variable       n     Mean     S.D.      Min      .25      Mdn      .75      Max
-------------------------------------------------------------------------------
 dbp_day       0        .        .        .        .        .        .        .
-------------------------------------------------------------------------------

. ranksum dbp_day, by (dip)

Two-sample Wilcoxon rank-sum (Mann-Whitney) test

         dip |      obs    rank sum    expected
-------------+---------------------------------
      dipper |       23       771.5       770.5
  non-dipper |       43      1439.5      1440.5
-------------+---------------------------------
    combined |       66        2211        2211

unadjusted variance     5521.92
adjustment for ties       -9.22
                     ----------
adjusted variance       5512.70

Ho: dbp_day(dip==dipper) = dbp_day(dip==non-dipper)
             z =   0.013
    Prob > |z| =   0.9893

.
```

```
.
. univar sbp_night, by (dip)

-> dip=dipper
                                        -------------- Quantiles --------------
Variable       n     Mean     S.D.      Min      .25      Mdn      .75      Max
-------------------------------------------------------------------------------
sbp_night     23   100.57    14.20    73.00    90.00    98.00   109.00   135.00
-------------------------------------------------------------------------------

-> dip=non-dipper
                                        -------------- Quantiles --------------
Variable       n     Mean     S.D.      Min      .25      Mdn      .75      Max
-------------------------------------------------------------------------------
sbp_night     43   114.74    19.78    86.00   102.00   114.00   124.00   173.00
-------------------------------------------------------------------------------

-> dip=.
                                        -------------- Quantiles --------------
Variable       n     Mean     S.D.      Min      .25      Mdn      .75      Max
-------------------------------------------------------------------------------
sbp_night      0        .        .        .        .        .        .        .
-------------------------------------------------------------------------------

. ranksum sbp_night, by (dip)

Two-sample Wilcoxon rank-sum (Mann-Whitney) test

         dip |      obs    rank sum    expected
-------------+---------------------------------
      dipper |       23         543       770.5
  non-dipper |       43        1668      1440.5
-------------+---------------------------------
    combined |       66        2211        2211

unadjusted variance     5521.92
adjustment for ties       -5.19
                      ----------
adjusted variance       5516.73

Ho: sbp_ni~t(dip==dipper) = sbp_ni~t(dip==non-dipper)
             z =  -3.063
    Prob > |z| =   0.0022

.
.
. univar dbp_night, by (dip)

-> dip=dipper
                                        -------------- Quantiles --------------
Variable       n     Mean     S.D.      Min      .25      Mdn      .75      Max
-------------------------------------------------------------------------------
dbp_night     23    60.91     8.54    51.00    55.00    58.00    68.00    89.00
-------------------------------------------------------------------------------

-> dip=non-dipper
                                        -------------- Quantiles --------------
Variable       n     Mean     S.D.      Min      .25      Mdn      .75      Max
-------------------------------------------------------------------------------
dbp_night     43    71.51    14.51    50.00    60.00    69.00    82.00   113.00
-------------------------------------------------------------------------------

-> dip=.
                                        -------------- Quantiles --------------
Variable       n     Mean     S.D.      Min      .25      Mdn      .75      Max
-------------------------------------------------------------------------------
dbp_night      0        .        .        .        .        .        .        .
-------------------------------------------------------------------------------

. ranksum dbp_night, by (dip)

Two-sample Wilcoxon rank-sum (Mann-Whitney) test

         dip |      obs    rank sum    expected
-------------+---------------------------------
      dipper |       23       543.5       770.5
  non-dipper |       43      1667.5      1440.5
-------------+---------------------------------
    combined |       66        2211        2211

unadjusted variance     5521.92
adjustment for ties      -11.30
                      ----------
adjusted variance       5510.62

Ho: dbp_ni~t(dip==dipper) = dbp_ni~t(dip==non-dipper)
             z =  -3.058
    Prob > |z| =   0.0022

.
.
```

```
. univar pwv, by (dip)

-> dip=dipper
                                        -------------- Quantiles --------------
Variable       n     Mean     S.D.      Min      .25      Mdn      .75      Max
-------------------------------------------------------------------------------
     pwv      23     6.71     1.81     4.70     5.20     6.20     7.80    11.00
-------------------------------------------------------------------------------

-> dip=non-dipper
                                        -------------- Quantiles --------------
Variable       n     Mean     S.D.      Min      .25      Mdn      .75      Max
-------------------------------------------------------------------------------
     pwv      41     6.80     1.51     4.70     5.80     6.40     6.90    10.20
-------------------------------------------------------------------------------

-> dip=.
                                        -------------- Quantiles --------------
Variable       n     Mean     S.D.      Min      .25      Mdn      .75      Max
-------------------------------------------------------------------------------
     pwv       1     8.90        .     8.90     8.90     8.90     8.90     8.90
-------------------------------------------------------------------------------

. ranksum pwv, by (dip)

Two-sample Wilcoxon rank-sum (Mann-Whitney) test

         dip |      obs    rank sum    expected
-------------+---------------------------------
      dipper |       23         704       747.5
  non-dipper |       41        1376      1332.5
-------------+---------------------------------
    combined |       64        2080        2080

unadjusted variance     5107.92
adjustment for ties       -5.85
                      ----------
adjusted variance       5102.07

Ho: pwv(dip==dipper) = pwv(dip==non-dipper)
             z =  -0.609
    Prob > |z| =   0.5425

.
.
. univar hs_crp, by (dip)

-> dip=dipper
                                        -------------- Quantiles --------------
Variable       n     Mean     S.D.      Min      .25      Mdn      .75      Max
-------------------------------------------------------------------------------
  hs_crp      23    14.09    32.86     0.30     1.60     4.60     7.80   152.50
-------------------------------------------------------------------------------

-> dip=non-dipper
                                        -------------- Quantiles --------------
Variable       n     Mean     S.D.      Min      .25      Mdn      .75      Max
-------------------------------------------------------------------------------
  hs_crp      43    14.73    54.36     0.20     2.20     4.40    10.40   359.80
-------------------------------------------------------------------------------

-> dip=.
                                        -------------- Quantiles --------------
Variable       n     Mean     S.D.      Min      .25      Mdn      .75      Max
-------------------------------------------------------------------------------
  hs_crp       0        .        .        .        .        .        .        .
-------------------------------------------------------------------------------

. ranksum hs_crp, by(dip)

Two-sample Wilcoxon rank-sum (Mann-Whitney) test

         dip |      obs    rank sum    expected
-------------+---------------------------------
      dipper |       23         784       770.5
  non-dipper |       43        1427      1440.5
-------------+---------------------------------
    combined |       66        2211        2211

unadjusted variance     5521.92
adjustment for ties       -1.96
                      ----------
adjusted variance       5519.96

Ho: hs_crp(dip==dipper) = hs_crp(dip==non-dipper)
             z =   0.182
    Prob > |z| =   0.8558

.
.
. tab metS_WC dip, col chi exact
```

```
+-------------------+
| Key               |
|-------------------|
|     frequency     |
| column percentage |
+-------------------+

           |          dip
   metS_WC |    dipper  non-dippe |     Total
-----------+----------------------+----------
         0 |         8          8 |        16 
           |     34.78      18.60 |     24.24 
-----------+----------------------+----------
         1 |        15         35 |        50 
           |     65.22      81.40 |     75.76 
-----------+----------------------+----------
     Total |        23         43 |        66 
           |    100.00     100.00 |    100.00 

          Pearson chi2(1) =   2.1355   Pr = 0.144
           Fisher's exact =                 0.227
   1-sided Fisher's exact =                 0.124

. 
. tab waistcat_fem2 dip, col chi exact

+-------------------+
| Key               |
|-------------------|
|     frequency     |
| column percentage |
+-------------------+

waistcat_f |          dip
       em2 |    dipper  non-dippe |     Total
-----------+----------------------+----------
     <80cm |         6          6 |        12 
           |     31.58      14.63 |     20.00 
-----------+----------------------+----------
     >80cm |        13         35 |        48 
           |     68.42      85.37 |     80.00 
-----------+----------------------+----------
     Total |        19         41 |        60 
           |    100.00     100.00 |    100.00 

          Pearson chi2(1) =   2.3299   Pr = 0.127
           Fisher's exact =                 0.169
   1-sided Fisher's exact =                 0.120

. 
. 
. 
. univar hs_crp, by (dip)

-> dip=dipper
                                        -------------- Quantiles --------------
Variable       n     Mean     S.D.      Min      .25      Mdn      .75      Max
-------------------------------------------------------------------------------
  hs_crp      23    14.09    32.86     0.30     1.60     4.60     7.80   152.50
-------------------------------------------------------------------------------

-> dip=non-dipper
                                        -------------- Quantiles --------------
Variable       n     Mean     S.D.      Min      .25      Mdn      .75      Max
-------------------------------------------------------------------------------
  hs_crp      43    14.73    54.36     0.20     2.20     4.40    10.40   359.80
-------------------------------------------------------------------------------

-> dip=.
                                        -------------- Quantiles --------------
Variable       n     Mean     S.D.      Min      .25      Mdn      .75      Max
-------------------------------------------------------------------------------
  hs_crp       0        .        .        .        .        .        .        .
-------------------------------------------------------------------------------

. ranksum hs_crp, by(dip)

Two-sample Wilcoxon rank-sum (Mann-Whitney) test

         dip |      obs    rank sum    expected
-------------+---------------------------------
      dipper |       23         784       770.5
  non-dipper |       43        1427      1440.5
-------------+---------------------------------
    combined |       66        2211        2211

unadjusted variance     5521.92
adjustment for ties       -1.96
                     ----------
adjusted variance       5519.96
```

```
Ho: hs_crp(dip==dipper) = hs_crp(dip==non-dipper)
             z =   0.182
    Prob > |z| =   0.8558

.
.
.
.
.
. tab hscrp_cat dip, col chi exact

+-------------------+
| Key               |
|-------------------|
|     frequency     |
| column percentage |
+-------------------+

Enumerating sample-space combinations:
stage 3:  enumerations = 1
stage 2:  enumerations = 2
stage 1:  enumerations = 0

           |          dip
 hscrp_cat |    dipper  non-dippe |     Total
-----------+----------------------+----------
        <1 |         2          6 |         8 
           |      8.70      13.95 |     12.12 
-----------+----------------------+----------
       1-3 |         4          9 |        13 
           |     17.39      20.93 |     19.70 
-----------+----------------------+----------
        >3 |        17         28 |        45 
           |     73.91      65.12 |     68.18 
-----------+----------------------+----------
     Total |        23         43 |        66 
           |    100.00     100.00 |    100.00 

          Pearson chi2(2) =   0.6071   Pr = 0.738
           Fisher's exact =                 0.788

.
.
.
.
. univar cap, by (dip)

-> dip=dipper
                                        -------------- Quantiles --------------
Variable       n     Mean     S.D.      Min      .25      Mdn      .75      Max
-------------------------------------------------------------------------------
     cap      23   117.09    16.46    91.00   105.00   116.00   131.00   153.00
-------------------------------------------------------------------------------

-> dip=non-dipper
                                        -------------- Quantiles --------------
Variable       n     Mean     S.D.      Min      .25      Mdn      .75      Max
-------------------------------------------------------------------------------
     cap      43   124.00    19.95    94.00   106.00   125.00   135.00   167.00
-------------------------------------------------------------------------------

-> dip=.
                                        -------------- Quantiles --------------
Variable       n     Mean     S.D.      Min      .25      Mdn      .75      Max
-------------------------------------------------------------------------------
     cap       1   130.00        .   130.00   130.00   130.00   130.00   130.00
-------------------------------------------------------------------------------

. ranksum cap, by(dip)

Two-sample Wilcoxon rank-sum (Mann-Whitney) test

         dip |      obs    rank sum    expected
-------------+---------------------------------
      dipper |       23       674.5       770.5
  non-dipper |       43      1536.5      1440.5
-------------+---------------------------------
    combined |       66        2211        2211

unadjusted variance     5521.92
adjustment for ties       -4.73
                     ----------
adjusted variance       5517.19

Ho: cap(dip==dipper) = cap(dip==non-dipper)
             z =  -1.292
    Prob > |z| =   0.1962

.
.
```

```
.
. univar aix, by (dip)

-> dip=dipper
                                        -------------- Quantiles --------------
Variable       n     Mean     S.D.      Min      .25      Mdn      .75      Max
-------------------------------------------------------------------------------
     aix      23    20.04    14.09    -1.00    11.00    19.00    28.00    46.00
-------------------------------------------------------------------------------

-> dip=non-dipper
                                        -------------- Quantiles --------------
Variable       n     Mean     S.D.      Min      .25      Mdn      .75      Max
-------------------------------------------------------------------------------
     aix      43    22.67    14.23    -1.00    11.00    23.00    32.00    60.00
-------------------------------------------------------------------------------

-> dip=.
                                        -------------- Quantiles --------------
Variable       n     Mean     S.D.      Min      .25      Mdn      .75      Max
-------------------------------------------------------------------------------
     aix       1    28.00        .    28.00    28.00    28.00    28.00    28.00
-------------------------------------------------------------------------------

. ranksum aix, by (dip)

Two-sample Wilcoxon rank-sum (Mann-Whitney) test

         dip |      obs    rank sum    expected
-------------+---------------------------------
      dipper |       23       709.5       770.5
  non-dipper |       43      1501.5      1440.5
-------------+---------------------------------
    combined |       66        2211        2211

unadjusted variance     5521.92
adjustment for ties       -6.57
                     ----------
adjusted variance       5515.35

Ho: aix(dip==dipper) = aix(dip==non-dipper)
             z =  -0.821
    Prob > |z| =   0.4114

.
.
.
. univar hr_day, by (dip)

-> dip=dipper
                                        -------------- Quantiles --------------
Variable       n     Mean     S.D.      Min      .25      Mdn      .75      Max
-------------------------------------------------------------------------------
  hr_day      23    80.04    11.26    55.00    72.00    80.00    90.00    98.00
-------------------------------------------------------------------------------

-> dip=non-dipper
                                        -------------- Quantiles --------------
Variable       n     Mean     S.D.      Min      .25      Mdn      .75      Max
-------------------------------------------------------------------------------
  hr_day      43    85.05     9.81    62.00    79.00    87.00    91.00   108.00
-------------------------------------------------------------------------------

-> dip=.
                                        -------------- Quantiles --------------
Variable       n     Mean     S.D.      Min      .25      Mdn      .75      Max
-------------------------------------------------------------------------------
  hr_day       0        .        .        .        .        .        .        .
-------------------------------------------------------------------------------

. ranksum hr_day, by (dip)

Two-sample Wilcoxon rank-sum (Mann-Whitney) test

         dip |      obs    rank sum    expected
-------------+---------------------------------
      dipper |       23         654       770.5
  non-dipper |       43        1557      1440.5
-------------+---------------------------------
    combined |       66        2211        2211

unadjusted variance     5521.92
adjustment for ties      -14.06
                     ----------
adjusted variance       5507.85

Ho: hr_day(dip==dipper) = hr_day(dip==non-dipper)
             z =  -1.570
    Prob > |z| =   0.1165

.
```

```
. univar hr_night, by (dip)

-> dip=dipper
                                        -------------- Quantiles --------------
Variable       n     Mean     S.D.      Min      .25      Mdn      .75      Max
-------------------------------------------------------------------------------
hr_night      23    73.26     9.87    49.00    67.00    74.00    81.00    92.00
-------------------------------------------------------------------------------

-> dip=non-dipper
                                        -------------- Quantiles --------------
Variable       n     Mean     S.D.      Min      .25      Mdn      .75      Max
-------------------------------------------------------------------------------
hr_night      43    76.63    10.87    60.00    69.00    74.00    83.00   110.00
-------------------------------------------------------------------------------

-> dip=.
                                        -------------- Quantiles --------------
Variable       n     Mean     S.D.      Min      .25      Mdn      .75      Max
-------------------------------------------------------------------------------
hr_night       0        .        .        .        .        .        .        .
-------------------------------------------------------------------------------

. ranksum hr_night, by (dip)

Two-sample Wilcoxon rank-sum (Mann-Whitney) test

         dip |      obs    rank sum    expected
-------------+---------------------------------
      dipper |       23       709.5       770.5
  non-dipper |       43      1501.5      1440.5
-------------+---------------------------------
    combined |       66        2211        2211

unadjusted variance     5521.92
adjustment for ties      -11.64
                     ----------
adjusted variance       5510.27

Ho: hr_night(dip==dipper) = hr_night(dip==non-dipper)
             z =  -0.822
    Prob > |z| =   0.4112

.
. *** rr and pos drop ****
. tab rr_abnorm dip,  col

+-------------------+
| Key               |
|-------------------|
|     frequency     |
| column percentage |
+-------------------+

           |          dip
 RR_abnorm |    dipper  non-dippe |     Total
-----------+----------------------+----------
         0 |        17         33 |        50
           |     73.91      76.74 |     75.76
-----------+----------------------+----------
         1 |         6         10 |        16
           |     26.09      23.26 |     24.24
-----------+----------------------+----------
     Total |        23         43 |        66
           |    100.00     100.00 |    100.00


. tab pos_drop dip, col

+-------------------+
| Key               |
|-------------------|
|     frequency     |
| column percentage |
+-------------------+

           |          dip
  pos_drop |    dipper  non-dippe |     Total
-----------+----------------------+----------
         0 |        16         40 |        56
           |     69.57      93.02 |     84.85
-----------+----------------------+----------
         1 |         7          3 |        10
           |     30.43       6.98 |     15.15
-----------+----------------------+----------
     Total |        23         43 |        66
           |    100.00     100.00 |    100.00


.
.
```

```
.
.
. **  Reverse dippers
. list no sbp_day sbp_night sbpchange sbpchange_percent if sbpchange<0

     +---------------------------------------------------+
     |  no   sbp_day   sbp_ni~t   sbpcha~e   sbpchan~t |
     |---------------------------------------------------|
  6. | 156       106        121        -15   -14.15094 |
 35. | 274       120        121         -1   -.8333333 |
 42. | 399       139        143         -4   -2.877698 |
 51. | 394       134        137         -3   -2.238806 |
     +---------------------------------------------------+

. tab reverse_dip

reverse_dip |      Freq.     Percent        Cum.
------------+-----------------------------------
          0 |         63       94.03       94.03
          1 |          4        5.97      100.00
------------+-----------------------------------
      Total |         67      100.00

.
. ******************************************************************************
. *************************          CRP vs WC       *************************
. ******************************************************************************
.
. tab hscrp_cat

  hscrp_cat |      Freq.     Percent        Cum.
------------+-----------------------------------
         <1 |          8       12.12       12.12
        1-3 |         13       19.70       31.82
         >3 |         45       68.18      100.00
------------+-----------------------------------
      Total |         66      100.00

.
.
. univar hs_crp, by (waistcat_fem2)

-> waistcat_fem2=<80cm
                                        -------------- Quantiles --------------
Variable       n     Mean     S.D.      Min      .25      Mdn      .75      Max
-------------------------------------------------------------------------------
  hs_crp      12    19.58    45.62     0.60     0.95     1.40     4.30   152.50
-------------------------------------------------------------------------------

-> waistcat_fem2=>80cm
                                        -------------- Quantiles --------------
Variable       n     Mean     S.D.      Min      .25      Mdn      .75      Max
-------------------------------------------------------------------------------
  hs_crp      48    14.34    51.38     0.20     3.30     4.85     9.10   359.80
-------------------------------------------------------------------------------

-> waistcat_fem2=.
                                        -------------- Quantiles --------------
Variable       n     Mean     S.D.      Min      .25      Mdn      .75      Max
-------------------------------------------------------------------------------
  hs_crp       6     5.68     4.79     0.60     1.30     4.80    11.20    11.40
-------------------------------------------------------------------------------

.
. ranksum hs_crp, by (waistcat_fem2)

Two-sample Wilcoxon rank-sum (Mann-Whitney) test

waistcat_f~2 |      obs    rank sum    expected
-------------+---------------------------------
       <80cm |       12         245         366
       >80cm |       48        1585        1464
-------------+---------------------------------
    combined |       60        1830        1830

unadjusted variance     2928.00
adjustment for ties       -0.98
                      ----------
adjusted variance       2927.02

Ho: hs_crp(waist~m2==<80cm) = hs_crp(waist~m2==>80cm)
             z =  -2.237
    Prob > |z| =   0.0253

.
.
.
. ******************************************************************************
. *************************      New CAP and AIX info       *******************
. ******************************************************************************
.
```

```
.
.
. **  NOTE ***
.
. ********* Layout as per Table 2 Central systolic blood pressure ***********
. ********* Compare normal popultaion with your cohort for 50th, 10th- 90th
.
.
. /*
> tab agecat2 sex
>
> . tab agecat2 sex
>
>   RECODE of |        Sex_M-1
>         age |    Female       Male |     Total
> ------------+----------------------+----------
>   <30 years |         7          0 |         7
> 30-39 years |        23          0 |        23
> 40-49 years |        22          3 |        25
> 50-59 years |         6          2 |         8
>   >60 years |         2          0 |         2
> ------------+----------------------+----------
>       Total |        60          5 |        65
>
>
> */
.
.
. *** For females only
. sum cap_30, d

                           cap_30
-------------------------------------------------------------
      Percentiles      Smallest
 1%           91             91
 5%           91            105
10%           91            106       Obs                   7
25%          105            112       Sum of Wgt.           7

50%          112                      Mean                111
                        Largest       Std. Dev.      12.62273
75%          118            112
90%          132            113       Variance       159.3333
95%          132            118       Skewness       .1138543
99%          132            132       Kurtosis       2.748267

. sum cap_30_39, d

                          cap_30_39
-------------------------------------------------------------
      Percentiles      Smallest
 1%           92             92
 5%           94             94
10%           94             94       Obs                  22
25%          105            102       Sum of Wgt.          22

50%        112.5                      Mean           116.5909
                        Largest       Std. Dev.      17.85833
75%          126            137
90%          138            138       Variance       318.9199
95%          138            138       Skewness       .9698672
99%          167            167       Kurtosis       3.968412

. sum cap_40_49, d

                          cap_40_49
-------------------------------------------------------------
      Percentiles      Smallest
 1%           96             96
 5%           98             98
10%           99             99       Obs                  23
25%          111            103       Sum of Wgt.          23

50%          130                      Mean           128.4348
                        Largest       Std. Dev.      19.81466
75%          142            153
90%          155            155       Variance       392.6206
95%          159            159       Skewness      -.0474365
99%          161            161       Kurtosis       2.015205

. sum cap_50_59, d

                          cap_50_59
-------------------------------------------------------------
      Percentiles      Smallest
 1%           93             93
 5%           93            102
10%           93            124       Obs                   7
25%          102            126       Sum of Wgt.           7

50%          126                      Mean           119.4286
```

```
                        Largest       Std. Dev.      15.61898
75%          130            126
90%          135            126       Variance       243.9524
95%          135            130       Skewness      -.8567329
99%          135            135       Kurtosis       2.141938

. sum cap_60, d

                           cap_60
-------------------------------------------------------------
      Percentiles      Smallest
 1%          155            155
 5%          155            160
10%          155              .       Obs                   2
25%          155              .       Sum of Wgt.           2

50%        157.5                      Mean              157.5
                        Largest       Std. Dev.      3.535534
75%          160              .
90%          160              .       Variance           12.5
95%          160            155       Skewness              0
99%          160            160       Kurtosis              1

.
. *** For males only - very small n though
. sum cap_40_49_male, d

                       cap_40_49_male
-------------------------------------------------------------
      Percentiles      Smallest
 1%          106            106
 5%          106            120
10%          106            128       Obs                   3
25%          106              .       Sum of Wgt.           3

50%          120                      Mean                118
                        Largest       Std. Dev.      11.13553
75%          128              .
90%          128            106       Variance            124
95%          128            120       Skewness      -.3193123
99%          128            128       Kurtosis            1.5

. sum cap_50_59_male, d

                       cap_50_59_male
-------------------------------------------------------------
      Percentiles      Smallest
 1%          105            105
 5%          105            118
10%          105            131       Obs                   3
25%          105              .       Sum of Wgt.           3

50%          118                      Mean                118
                        Largest       Std. Dev.            13
75%          131              .
90%          131            105       Variance            169
95%          131            118       Skewness              0
99%          131            131       Kurtosis            1.5

.
. ******************************************************************************
.
. tab agecat3

  RECODE of |
        age |      Freq.     Percent        Cum.
------------+-----------------------------------
  <30 years |          7       10.45       10.45
30-49 years |         48       71.64       82.09
  >50 years |         12       17.91      100.00
------------+-----------------------------------
      Total |         67      100.00

.
. /*
> . tab agecat3 sex_m1
>
>   RECODE of |        Sex_M-1
>         age |    Female       Male |     Total
> ------------+----------------------+----------
>   <30 years |         7          0 |         7
> 30-49 years |        45          3 |        48
>   >50 years |         8          2 |        10
> ------------+----------------------+----------
>       Total |        60          5 |        65
>
>
> */
.
. sum pp_30, d
```

```
                            pp_30
-------------------------------------------------------------
      Percentiles      Smallest
 1%           28             28
 5%           28             30
10%           28             30       Obs                   7
25%           30             35       Sum of Wgt.           7

50%           35                      Mean           34.57143
                        Largest       Std. Dev.      5.349677
75%           39             35
90%           42             38       Variance       28.61905
95%           42             39       Skewness       .0732845
99%           42             42       Kurtosis       1.497148

. sum pp_30_49, d

                          pp_30_49
-------------------------------------------------------------
      Percentiles      Smallest
 1%           22             22
 5%           27             25
10%           28             27       Obs                  48
25%           31             28       Sum of Wgt.          48

50%           37                      Mean           37.85417
                        Largest       Std. Dev.      8.744578
75%           42             51
90%           49             56       Variance       76.46764
95%           56             58       Skewness       .7763504
99%           63             63       Kurtosis       3.433877

. sum pp_50, d

                            pp_50
-------------------------------------------------------------
      Percentiles      Smallest
 1%           31             31
 5%           31             33
10%           33             33       Obs                  12
25%         34.5             36       Sum of Wgt.          12

50%           39                      Mean              40.75
                        Largest       Std. Dev.      7.387028
75%         47.5             47
90%           49             48       Variance       54.56818
95%           54             49       Skewness       .3498875
99%           54             54       Kurtosis       1.891265

.
. sum aix_30, d

                           aix_30
-------------------------------------------------------------
      Percentiles      Smallest
 1%           -1             -1
 5%           -1              5
10%           -1              5       Obs                   7
25%            5              6       Sum of Wgt.           7

50%            6                      Mean           7.714286
                        Largest       Std. Dev.      5.707138
75%           13              6
90%           16             10       Variance       32.57143
95%           16             13       Skewness       .0209892
99%           16             16       Kurtosis       2.090342

. sum aix_30_49, d

                          aix_30_49
-------------------------------------------------------------
      Percentiles      Smallest
 1%           -1             -1
 5%            0             -1
10%            1              0       Obs                  48
25%         11.5              0       Sum of Wgt.          48

50%           21                      Mean           22.39583
                        Largest       Std. Dev.      14.08899
75%         31.5             45
90%           42             45       Variance       198.4996
95%           45             46       Skewness       .2935826
99%           60             60       Kurtosis       2.700586

. sum aix_50, d

                           aix_50
-------------------------------------------------------------
      Percentiles      Smallest
 1%            6              6
 5%            6             19
```

```
10%           19             20       Obs                  12
25%           20             20       Sum of Wgt.          12

50%           26                      Mean           27.91667
                        Largest       Std. Dev.      12.17642
75%           35             30
90%           45             40       Variance       148.2652
95%           50             45       Skewness       .2766875
99%           50             50       Kurtosis       2.658772

.
. *****************************************************************************
.
.
. bysort agecat2: tabstat pwv, stats (n mean sd p50 p25 p75)

------------------------------------------------------------------------------------------------------------------------
-------------------------------
-> agecat2 = <30 years

    variable |         N      mean        sd       p50       p25       p75
-------------+------------------------------------------------------------
         pwv |         7  5.614286  .5398412       5.9         5         6
--------------------------------------------------------------------------

------------------------------------------------------------------------------------------------------------------------
-------------------------------
-> agecat2 = 30-39 years

    variable |         N      mean        sd       p50       p25       p75
-------------+------------------------------------------------------------
         pwv |        22  6.531818  1.329657      6.35       5.5       7.6
--------------------------------------------------------------------------

------------------------------------------------------------------------------------------------------------------------
-------------------------------
-> agecat2 = 40-49 years

    variable |         N      mean        sd       p50       p25       p75
-------------+------------------------------------------------------------
         pwv |        25     7.076  1.628721       6.7       6.1       7.8
--------------------------------------------------------------------------

------------------------------------------------------------------------------------------------------------------------
-------------------------------
-> agecat2 = 50-59 years

    variable |         N      mean        sd       p50       p25       p75
-------------+------------------------------------------------------------
         pwv |        10      7.21  2.074421      6.55       5.8       9.3
--------------------------------------------------------------------------

------------------------------------------------------------------------------------------------------------------------
-------------------------------
-> agecat2 = >60 years

    variable |         N      mean        sd       p50       p25       p75
-------------+------------------------------------------------------------
         pwv |         1      10.2         .      10.2      10.2      10.2
--------------------------------------------------------------------------

.
end of do-file

. log close
      name:  <unnamed>
       log:  /Users/grethenothling/Box Sync/Stats Sync/Megan B/Output/March 2017/Megan B cross-sectional analysis
10_03_2017.log
  log type:  text
 closed on:  10 Mar 2017, 15:56:49
------------------------------------------------------------------------------------------------------------------------
-------------------------------
```
